# A Proteomic View at the Biochemistry of Syntrophic Butyrate Oxidation in *Syntrophomonas wolfei*

**DOI:** 10.1371/journal.pone.0056905

**Published:** 2013-02-26

**Authors:** Alexander Schmidt, Nicolai Müller, Bernhard Schink, David Schleheck

**Affiliations:** 1 Department of Biology, University of Konstanz, Konstanz, Germany; 2 Konstanz Research School Chemical Biology (KoRS-CB), University of Konstanz, Konstanz, Germany; Institut Pasteur Paris, France

## Abstract

In syntrophic conversion of butyrate to methane and CO_2_, butyrate is oxidized to acetate by secondary fermenting bacteria such as *Syntrophomonas wolfei* in close cooperation with methanogenic partner organisms, e.g., *Methanospirillum hungatei*. This process involves an energetically unfavourable shift of electrons from the level of butyryl-CoA oxidation to the substantially lower redox potential of proton and/or CO_2_ reduction, in order to transfer these electrons to the methanogenic partner *via* hydrogen and/or formate.

In the present study, all prominent membrane-bound and soluble proteins expressed in *S. wolfei* specifically during syntrophic growth with butyrate, in comparison to pure-culture growth with crotonate, were examined by one- and two-dimensional gel electrophoresis, and identified by peptide fingerprinting-mass spectrometry. A membrane-bound, externally oriented, quinone-linked formate dehydrogenase complex was expressed at high level specifically during syntrophic butyrate oxidation, comprising a selenocystein-linked catalytic subunit with a membrane-translocation pathway signal (TAT), a membrane-bound iron-sulfur subunit, and a membrane-bound cytochrome. Soluble hydrogenases were expressed at high levels specifically during growth with crotonate. The results were confirmed by native protein gel electrophoresis, by formate dehydrogenase and hydrogenase-activity staining, and by analysis of formate dehydrogenase and hydrogenase activities in intact cells and cell extracts. Furthermore, constitutive expression of a membrane-bound, internally oriented iron-sulfur oxidoreductase (DUF224) was confirmed, together with expression of soluble electron-transfer flavoproteins (EtfAB) and two previously identified butyryl-CoA dehydrogenases.

Our findings allow to depict an electron flow scheme for syntrophic butyrate oxidation in *S. wolfei*. Electrons derived from butyryl-CoA are transferred through a membrane-bound EtfAB:quinone oxidoreductase (DUF224) to a menaquinone cycle and further *via* a *b*-type cytochrome to an externally oriented formate dehydrogenase. Hence, an ATP hydrolysis-driven proton-motive force across the cytoplasmatic membrane would provide the energy input for the electron potential shift necessary for formate formation.

## Introduction

Fermentation of butyrate to methane and CO_2_ is catalyzed by fatty acid-oxidizing bacteria in syntrophic cooperation with hydrogen-scavenging, methanogenic partner organisms, e.g., by *Syntrophomonas wolfei* in cooperation with *Methanospirillum hungatei*. Under these conditions, the butyrate-oxidizing bacteria can gain energy in the range of approximately −20 kJ per mol of butyrate oxidized [1], which is just sufficient to support microbial growth [2]. However, the biochemical mechanism of syntrophic butyrate oxidation by *S. wolfei* has not yet been resolved [3].

Fermentation of butyrate by *S. wolfei* involves hydrogen formation by reduction of protons (or formate formation by reduction of CO_2_) with electrons released in the *beta*-oxidations of butyrate, while the methanogenic partner has to maintain a very low hydrogen partial pressure to keep the overall degradative reactions thermodynamically favorable, hence, to allow for butyrate oxidation to two acetate and simultaneous energy conservation [1], [4], [5]. Production of hydrogen (or formate) with electrons derived from butyrate oxidation is energetically unfavourable: the midpoint potential of the proton/hydrogen couple is raised to −300 to −250 mV if the methanogenic partner organisms keep the hydrogen concentration below 10^−4^ atm hydrogen [1], [2]. This level can just be met by electrons delivered *via* NADH (E^0′^ = −320 mV [6]), and NAD^+^ is indeed the electron acceptor in the second oxidation step in the butyrate pathway, from 3-hydroxybutyryl-CoA to acetoacetyl-CoA (E^0′^ = −250 mV [7]) catalysed by an NAD^+^-dependent 3-hydroxybutyryl-CoA dehydrogenase [8], [9]. However, two electrons are released at a much higher redox potential in the first oxidation step of the butyrate pathway, from butyryl-CoA to crotonyl-CoA (E^0′^ = −125 mV/−10 mV [7], [10]). This reaction is catalysed by butyryl-CoA dehydrogenase that passes the electrons on to electron-transfer flavoproteins (EtfAB). To release these electrons as hydrogen or formate, it is assumed that *S. wolfei* has to sacrifice part of the energy that is conserved as ATP in the acetate kinase reaction into a ‘reversed electron transport’ [11].

To understand how *S. wolfei* couples the oxidation of butyrate with hydrogen/formate formation, the genome sequence has been thoroughly analyzed and annotated [12]. The *S. wolfei* genome encodes five gene clusters for formate dehydrogenases (FDH-1 – FDH-5; numbering according to Sieber *et al.* [12]): two of these (FDH-2 and FDH-4) are predicted to be externally oriented (e.g., the catalytic subunit genes encode TAT membrane-translocation pathway signals) and linked to the menaquinone cycle *via* a membrane-bound *b*-type cytochrome (co-encoded in the same gene clusters), whereas the other three (FDH-1, FDH-3, FDH-5) are predicted to be cytoplasmatically oriented and linked to NADH *via* NADH:quinone oxidoreductases (co-encoded in the same gene clusters) [12]. Furthermore, three hydrogenases (HYD-1 - HYD-3) were predicted [12], one (HYD-2) externally oriented and linked to the menaquinone cycle *via* a *b*-type cytochrome (co-encoded in the same gene cluster), and one (HYD-1) homologous to the electron-confurcating hydrogenase complex in *Thermotoga maritima* [13]; the third hydrogenase catalytic subunit (HYD-3) is encoded solitarily in the genome, i.e., is not encoded in a gene cluster with electron transfer-component genes.

Experimental evidence for the involvement of a proton gradient and of ATPase activity in the predicted reversed electron tranport was obtained with intact cell suspensions [11], and it was hypothesized that menaquinone-7 could play an essential role in this reaction [11]. Furthermore, we recently enriched a membrane-associated NADH:acceptor oxidoreductase activity from butyrate-grown *S. wolfei* cells which was identified to derive from gene (IMG locus tag) Swol_1018 annotated as NADH-binding subunit gene of the HYD-1 hydrogenase complex (see above), i.e., located in the Swol_1017-19 gene cluster. Interestingly, the other two components of the HYD-1 complex, Swol_1017 and Swol_1019, were co-purified (and co-identified) with the NADH:acceptor oxidoreductase activity (Swol_1018) from butyrate-grown *S. wolfei*. Additionally, the catalytic subunit of formate dehydrogenase FDH-1 (see above) was co-purifed with this complex (and co-identified), represented by genes Swol_0785–86 (selenocysteine-linked FDH-1 subunit) [14]. The protein complex was interpreted to likely represent a membrane-associated, internally oriented NADH:hydrogenase/formate-dehydrogenase complex that may generate hydrogen and/or formate [14]. In the same study, we purified the butyryl-CoA dehydrogenase (BCD) activity of butyrate-grown *S. wolfei*, which turned out to be a complex of two BCDs (Swol_1933 and _2052) and a predicted membrane-associated FeS-containing reductase (cystein-rich reductase, DUF224) (Swol_0698) which could transfer electrons to the membrane-associated NADH:hydrogenase/formate-dehydrogenase complex [14].

In the present study, we used differential proteomics to identify all abundant proteins expressed specifically during syntrophic growth of *S. wolfei* with butyrate. Protein extracts (membrane and soluble fraction) of syntrophically butyrate-grown and of pure culture crotonate-grown *S. wolfei* cells were compared by denaturing polyacrylamid-gel electrophoresis (1D- and 2D-PAGE), and all proteins of interest were excised and identified by peptide fingerprinting-mass spectrometry (PF-MS). Furthermore, we used anoxic Blue-Native PAGE and formate dehydrogenase and hydrogenase-activity staining, and identified all stained protein bands by PF-MS. The results combined with enzyme tests to confirm the function and location of specific formate dehydrogenases and hydrogenase activities derived a much more detailed picture of the reversed electron transport in syntrophic butyrate oxidation by *S. wolfei*.

## Results

### Proteins identified by peptide fingerprinting-mass spectrometry in the membrane fraction of *S. wolfei* cells

**Figure 1:**
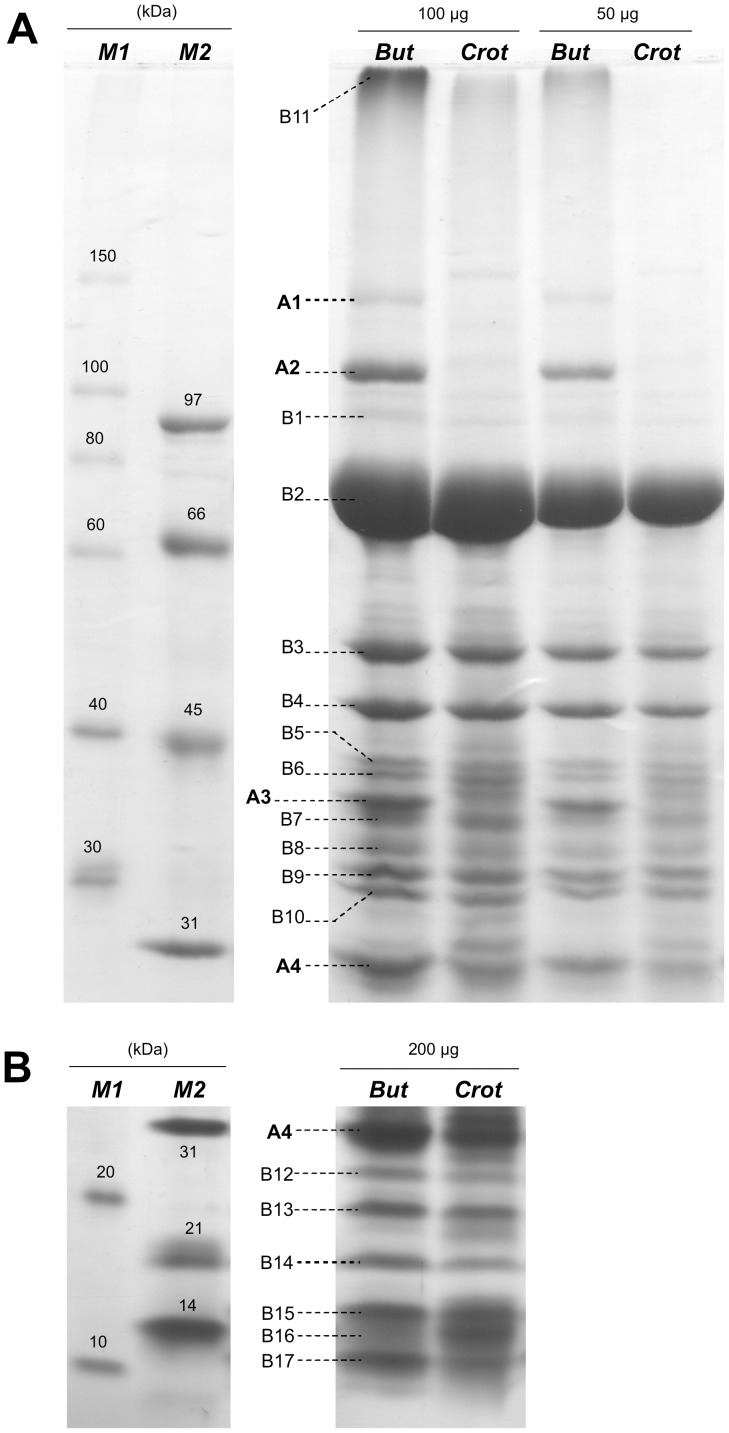
Representative SDS-PAGE gels of solubilised membrane proteins of butyrate- or crotonate-grown *S. wolfei* cells. The protein banding pattern in the high (A) and low (B) molecular mass range, and the labelling of the 21 discrete protein bands (bands A1–A4, and B1–B17) that were excised and identified by PF-MS (see Table 1), is shown. Washed membranes of *S. wolfei* were solubilised and separated using gradient gels (see text). Four bands were visible solely if butyrate-grown *S. wolfei* was analyzed (bands A1–A4), whereas 17 bands were visible for both, butyrate- and crotonate-grown *S. wolfei* (bands B1–B17). Legend: *But* and *Crot*, membrane proteins from butyrate- and crotonate-grown *S. wolfei* cells, respectively, with 100 µg and 50 µg (A) or 200 µg (B) total protein loaded; *M1* and *M2*, two different preparations of molecular mass-marker proteins were used for comparison (*M1* = Roti-Mark 10–150; *M2 = *BioRad Low-Range);

Isolated membrane fragments derived from *S. wolfei* cells grown with butyrate or crotonate were solubilized with SDS or dodecylmaltoside and the solubilized proteins separated by 1D-SDS-PAGE (see Material and Methods). A pattern of more than 20 prominent protein bands was obtained when gradient gels were used (Fig. 1AB). Four of these bands were more prominent, or observed exclusively, in extracts of butyrate-grown cells (band A1–A4 in Fig. 1A), and therefore are suggested to represent butyrate-induced proteins. The other prominent bands were visible at similar intensity in both, butyrate- and crotonate-grown cells (B1–B17 in Fig. 1AB), and these proteins were considered to be constitutively expressed. The twenty most prominent bands were excised (as indicated in Fig. 1AB) and submitted to peptide fingerprinting-mass spectrometry (PF-MS) and database-searching (see Material and Methods) against the genome sequence of *S. wolfei* (Table 1). A band observed at the start of the SDS-PAGE separation gel (band B11 in Fig. 1A) was also excised and submitted to PF-MS.

**Table 1:** Identifications obtained by peptide fingerprinting-mass spectrometry for all abundant solubilised membrane proteins of *S. wolfei* separated by SDS-PAGE (see Fig. 1AB), assorted into groups of protein bands that, (A) were observed specifically for syntrophically butyrate-grown *S. wolfei*, and (B) protein bands that were observed for both *S. wolfei* cells grown with butyrate and crotonate.

Protein band (no.)	Massa (kDa)				Identification			
		Gene locus tag (Swol_)	Signal peptide^b^	Trans-membrane helices^c^	Annotation	Predicted mass (Da)	Score^d^	Sequence coverage^d^ (%)
A2	110	0799	―	―	formate dehydrogenase subunit, molybdopterin-binding (FDH-2)	95,579	1118	67
		0800	TAT	―	formate dehydrogenase subunit, selenocysteine-containing (FDH-2)	25,870	212	50
A4	30	0798	―	(yes)^f^	formate dehydrogenase iron-sulfur subunit (FDH-2)	29,955	227	59
		2432	SEC	yes	ABC-type transport substrate-binding protein (metal uptake)	30,098	460	50
A3	35	1630	SEC	yes	ABC-type transport permease protein (tungstate uptake)	38,493	1649	62
A1	140^e^	0143	SEC	yes	hypothetical outer membrane protein (invasin/intimin/lectin-like)	141,664	1221	29
B3	50	2384	―	―	ATP synthase, proton/sodium translocating, F1 *alpha*-subunit	54,508	454	37
B4	45	2382	―	―	ATP synthase, proton/sodium translocating, F1 *beta*-subunit	51,311	1033	91
B9	30	2383	―	―	ATP synthase, proton/sodium translocating, F1 *gamma*-subunit	33,080	1041	65
B12	25	2388	―	yes	ATP synthase, proton/sodium translocating, F0F1-type, F0 subunit *a*	25,111	198	43
B13	20	2385	―	―	ATP synthase, proton/sodium translocating, F1 *delta*-subunit	20,349	226	56
B14	20	2386	―	yes	ATP synthase, proton/sodium translocating, F0F1-type, F0 subunit *b*	18,937	69	28
B15	15	2381	―	―	ATP synthase, proton/sodium translocating, F1 *epsilon*-subunit	15,201	364	61
B1	100	1161	SEC	yes	uncharacterized integral membrane protein (UPF0182)	106,056	276	24
B7	35	0698	―	yes	iron-sulfur membrane protein (cysteine-rich DUF224 protein)	81,417^g^	480	37
B2	70^e^	0133	(SEC)^f^	―	hypothetical outer-membrane protein (predicted S-layer protein)	78,098	3301	77
B8	32	0413	SEC	―	conserved hypothetical lipoprotein	42,747	883	55
B16	14	0671	―	―	hypothetical protein	16,690	191	54
		1946	―	―	hypothetical protein	12,368	153	41
B17	12	1244	―	―	hypothetical protein (sequence-specific DNA-binding)	13,576	385	67
B5	40	2556	SEC	yes	ABC-type transport substrate-binding protein (amino acid uptake)	41,858	1229	66
B6	38	0405	SEC	yes	ABC-type transport substrate-binding protein (carbohydrate uptake)	48,290	859	55
B10	28	0331	SEC	yes	TRAP-type transport substrate-binding protein (COG1638)	41,235	220	35
		0423	SEC	―	ABC-type transport substrate-binding protein (carbohydrate uptake)	38,076	135	32
B11	n/a^h^	1425	yes	yes	protein export membrane protein SecD	43,254	238	26
		0091	―	yes	formate/nitrite-family transporter	30,939	120	19
		0698	―	yes	iron-sulfur membrane protein (cysteine-rich DUF224 protein)	81,417	80	13
		0797	―	yes	cytochrome-*b* subunit of formate dehydrogenase (FDH-2)	25,730	43	11
		0799	―	―	formate dehydrogenase subunit, molybdopterin-binding (FDH-2)	95,579	34	8
		0800	TAT	―	formate dehydrogenase subunit, selenocysteine-containing (FDH-2)	25,870	31	8

a , apparent mass of the protein on 1D-SDS-PAGE.

b , signal for (pre)protein secretion *via* Sec-dependent (SEC) or TAT-translocation (TAT) pathway predicted by SignalP and PRED-TAT (probab. >0.6).

c , transmembrane helix/helices predicted using TMHMM 2.0 and PRED-TAT (probab. >0.6).

d , score and sequence coverage of the peptide fingerprint match as indicated by the MASCOT-search engine.

e , protein bands were only observed if the membranes were solubilised with SDS, but not when dodecylmaltosid was used (see text).

f , predictions <0.6 probability for secretion-signals or transmembrane helices, however the ortholog proteins in the databases exhibit valid prediction(s).

g , discrepancy between observed and predicted molecular mass is attributed to a loss of the membrane anchor of the proteins (see text).

h , not applicable; the PF-MS derived of a protein band that was retained at the start of the separation gel (see Fig. 1).

The most prominent butyrate-induced protein, band A2 at appr. 110 kDa molecular mass (Fig. 1A), yielded a peptide fingerprint that validly identified two genes in the genome of *S. wolfei*, Swol_0800 and Swol_0799, which are annotated to conjointly encode a selenocysteine-linked formate dehydrogenase (FDH) catalytic subunit (appr. 26 plus 95 kDa) (Table 1). These genes were previously attributed by Sieber *et al.* [12] as candidates for a membrane-bound, externally oriented formate dehydrogenase in *S. wolfei* (FDH-2 according to Sieber *et al.* [12]), since Swol_0800 encodes a predicted twin-arginine translocation (TAT) pathway signal (reliability score: 0.95) for active translocation of the folded protein across the cytoplasmic membrane. Co-encoded in the gene cluster Swol_0799-0800 is also a membrane-bound iron-sulfur subunit (30 kDa) candidate gene (Swol_0798) and a membrane-bound cytochrome *b* (26 kDa) candidate gene (Swol_0797) [12]. The FDH iron-sulfur subunit gene (Swol_0798) was validly identified by the peptide fingerprint obtained from a second prominent, butyrate-induced, membrane protein band, band A4 (at appr. 30 kDa in Fig. 1A; Table 1). The gene for cytochrome (Swol_0797) however, was not matched by any band excised in the appropriate molecular mass range (20–30 kDa; e.g., not by bands B12–B17 in Fig. 1B), but was attributed by the fingerprint of band B11 (Fig. 1A) that contained all proteins not well mobilized during SDS-PAGE (see Table 1). However, the identity and strong expression of the FDH-2 complex (Swol_0798-800) exclusively in the membrane of butyrate-grown *S. wolfei* cells was confirmed in comparison to crotonate-grown cells when the native complex was solubilized using dodecylmaltoside, and separated by Blue-Native PAGE from the solubilized ATP synthase complex (see below), followed by second-dimension denaturing SDS-PAGE and identification of the same two subunits by PF-MS (see Supplemental information, Fig. S1). Finally, the third prominent butyrate-induced membrane protein, band A3 (Fig. 1), was attributed to a gene annotated to encode an ABC-transport permease for the uptake of tungstate (Swol_1630; Table 1). The fourth butyrate-induced protein, band A1, was attributed to a ‘conserved hypothetical gene’ (Swol_0143; Table 1), e.g., in *Clostridium* species, that could encode a cell-adhesion and/or sugar-binding protein. Interestingly, this protein was not observed on gels when dodecylmaltoside instead of SDS was used to solubilize the membranes (see Fig. S2 in the Supplemental information).

Among the constitutively expressed membrane proteins identified (bands B1–B17 in Fig. 1AB and Table 1), the fingerprint of band B7 identified Swol_0698, which encodes a membrane-bound FeS-oxidoreductase protein, but band B7 was observed at lower molecular mass (appr. 35 kDa in Fig. 1) than predicted by the Swol_0698 gene sequence (appr. 81 kDa, Table 1). The same observation was made previously when this gene was identified for the first time to be expressed in *S. wolfei* during syntrophic butyrate oxidation [14]. The membrane-bound FeS-containing oxidoreductase Swol_0698 in conjunction with EtfAB is presumed to link the electron flow from butyryl-CoA dehydrogenase to the membrane [12], [14] and the Swol_0698 gene is clustered with EtfB and EtfA genes (Swol_0696-97 [12], [14]) whose co-expression was confirmed also in this study (see below).

Seven of the other bands of constitutively expressed membrane proteins identified were attributed to the proton- or sodium-driven ATP synthase complex (B3, B4, B9 in Fig. 1A; B12 - 15 in Fig. 1B) that is encoded by a single ATP synthase gene cluster in the *S. wolfei* genome [12] (Swol_2381-2388, see Table 1). Moreover, three substrate-binding component genes of ABC-transport systems were identified (band B5, Swol_2556; band B6, Swol_0405; band B10, Swol_0423) in addition to the one already identified (see above, band A4, Swol_2432), and a substrate-binding protein of a TRAP-type transport system (band B10, Swol_0331). Hypothetical genes were attributed to band B1 (Swol_1161; putative membrane-integral protein gene, UPF0182), band B8 (Swol_0413; ‘conserved hypothetical lipoprotein’, e.g. in *Clostridium* species), and to the minor bands B16 and B17 (see Table 1). The peptide fingerprint of band B11, i.e. all membrane proteins that were not well mobilised during SDS-PAGE, were attributed (see Table 1) to Swol_1425 for a protein-export SecD-family membrane protein and to Swol_0091 for a formate/nitrite-transporter (FNT) family protein, each with a score>100; weaker matches (score<100) were obtained to the gene for the membrane-bound FeS-containing oxidoreductase (see above, Swol_0698), to the membrane-bound cytochrome subunit gene (see above, Swol_0797), and to the selenocysteine-linked formate dehydrogenase catalytic subunit (see above, Swol_0800-799) (Table 1).

Notably, the most abundant protein observed in the membrane fraction of both, butyrate- and crotonate-grown *S. wolfei* cells, was represented by band B2 (see Fig. 1A), which identified yet another ‘conserved hypothetical protein’ gene, Swol_0133 (Table 1), that is widespread within the phylum *Firmicutes*. Interestingly, this protein (Swol_0133) was not observed on gels when dodecylmaltoside instead of SDS was used to solubilize the membranes (see Fig. S2 in the Supporting Information). No valid predictions of protein-secretion signals and/or transmembrane helices were obtained for Swol_0133, however, valid predictions of SEC-secretion signals were obtained if the orthologous genes were analyzed (not shown). Furthermore, no conserved domain could be detected in Swol_0133 other than an N-terminal domain of certain outer-membrane copper amine oxidases, cell-wall hydrolases, and amidases (IPR012854), but at the C-terminal end of the Swol_0133 sequence (10% alignment length). However, the Swol_0133 sequence showed weak homology (27% identity) to surface-layer (S-layer) glycoproteins, e.g., to the SatA precursor of *Aneurinibacillus thermoaerophilus* (AAS44591 [15]) (see Discussion).

### Proteins identified by peptide fingerprinting-mass spectrometry in the soluble fraction of *S. wolfei* cells

**Figure 2:**
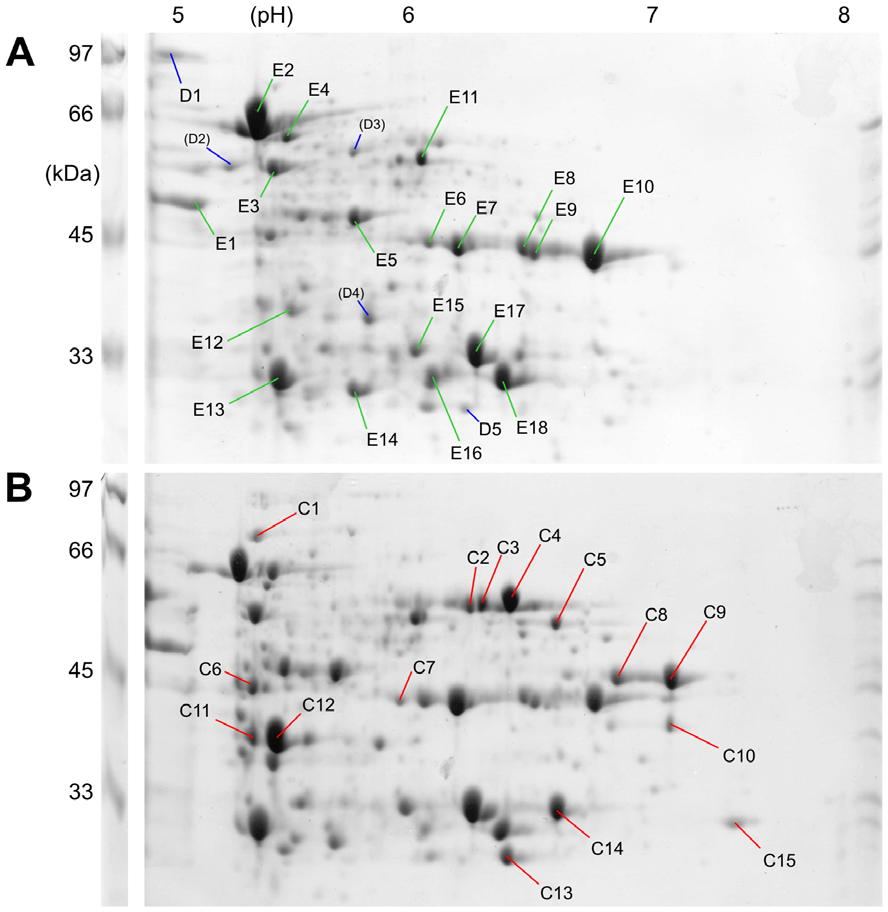
Representative two-dimensional IEF/SDS-PAGE gels of soluble proteins of butyrate- or crotonate-grown *S. wolfei* cells. All major protein spots on the gel from butyrate-grown cells (A), and the proteins spots that appeared to be differentially expressed in crotonate-grown cells (B), were excised and identified by PF-MS (see Table 2).

Proteins in the soluble fraction of butyrate- and crotonate-grown cells were separated by 2D-PAGE, firstly, in the pH-range 3–10 for isoelectric focussing (not shown) and secondly, in the pH-range 5–8 (Fig. 2). A total of 54 spots (including replicates) were excised and identified by PF-MS, and sorted (Table 2) into proteins that appeared either exclusively in extracts of butyrate-grown cells (spots D1–D5), in both extracts (spots E1–E18; excised from the butyrate-gel), or exclusively in extracts of crotonate-grown cells (spots C1–C15).

**Table 2:** Identifications obtained by peptide fingerprinting-mass spectrometry for the protein spots excised from 2D-gels of soluble proteins in *S. wolfei* (see Fig. 2), assorted into groups of protein spots that were observed uniquely on gels from butyrate-grown *S. wolfei* (D), on gels from both butyrate- and crotonate-grown cells (E), and spots observed uniquely on gels from crotonate-grown cells (C).

Protein band (no.)	Massa (kDa)	pI^a^		Identification				
		(pH)	Gene locus tag (Swol_)	Annotation	Predicted mass^b^ (Da)	Pred. pI^b^ (pH)	Score^c^	Sequence coverage^c^ (%)
D1	97	5	2054	putative flavoprotein (uncharacterized flavoprotein [COG0426] domain fused with NAD(P)H-accepting domain of nitrite reductase [COG1251])	98,400	4.64	1193	47
D5	25	6	2452	carbonic anhydrase (COG0288)	25,996	6.09	544	37
E2	65	5.5	1933	butyryl-CoA dehydrogenase (COG1960)	67,830	4.75	877	45
E4	65	5.5	2052	butyryl-CoA dehydrogenases (COG1960)	67,931	4.84	805	40
E11	50	6	1932	acetyl-CoA hydrolase/transferase (COG0427)	49,841	5.62	818	38
E6	45	6	0768	acetate kinase (COG0282)	43,420	5.97	660	47
E7	45	6.5	0768	acetate kinase (COG0282)	43,420	5.97	754	43
E8	45	6.5	1934	acetyl-CoA acetyltransferase (COG0183)	41,283	6.31	1213	66
E9	45	6.5	1934	acetyl-CoA acetyltransferase (COG0183)	41,283	6.31	894	57
E10	45	7	1934	acetyl-CoA acetyltransferase (COG0183)	41,283	6.31	1599	55
E12	35	5.5	0767	phosphotransacetylase (COG0280)	34,553	4.94	592	58
E16	30	6.0	1935	3-hydroxybutyryl-CoA dehydrogenase (COG1250)	29,762	5.91	610	67
E18	30	6.5	1935	3-hydroxybutyryl-CoA dehydrogenase (COG1250)	29,762	5.91	803	68
E14	30	5.7	1936	3-hydroxybutyryl-CoA dehydratase (COG1024)	27,945	5,03	506	54
E13	30	5.5	0696	electron transfer flavoprotein, *beta*-subunit (EtfB) (COG2086)	26,280	4.81	754	56
E17	33	6.3	0697	electron transfer flavoprotein, *alpha*-subunit (EtfA) (COG2025)	33,043	5,81	465	48
E5	45	5.7	1018	NADH:ubiquinone oxidoreductase (COG1894)	43,921	5.09	1161	47
E3	50	5.5	2384	ATP synthase, proton/sodium translocating, F1 *alpha*-subunit	54,509	4.86	743	41
E1	48	5	2382	ATP synthase, proton/sodium translocating, F1 *beta*-subunit	51,312	4.61	496	42
E15	33	6	0007	vitamin B6 biosynthesis protein	31,640	5.48	148	27
C2	55	6.3	1017	hydrogenase, Fe-only catalytic subunit (HYD-1)	62,989	5.94	1070	43
C3	55	6.3	1017	hydrogenase, Fe-only catalytic subunit (HYD-1)	62,989	5.94	271	21
			2436	hydrogenase, Fe-only catalytic subunit (HYD-3)	61,251	6.11	251	19
C4	55	6.5	2436	hydrogenase, Fe-only catalytic subunit (HYD-3)	61,251	6.11	140	23
C6	45	5.5	0412	2-hydroxyacyl-CoA dehydratase/benzoyl-CoA reductase [4Fe4S] subunit (COG1775)	47,404	4.83	716	52
C7	33	6.5	2126	butyryl-CoA dehydrogenase (COG1960)	41,155	5,32	625	58
			0768	acetate kinase (COG0282)	43,420	5.97	486	29
C10	43	7	0675	acetyl-CoA acetyltransferase (COG0183)	42,134	6.76	959	54
C15	31	7.5	2030	3-hydroxybutyryl-CoA dehydrogenase (COG1250)	29,998	7.25	344	59
C14	30	6.5	0435	3-hydroxybutyryl-CoA dehydrogenase (COG1250)	22,133	5.44	442	70
C5	50	6.5	0436	acetyl-CoA hydrolase/transferase (COG0427)	49,951	6.03	669	49
C8	45	6.7	unidentified^d^	-	-	-	-	-
C9	40	7	unidentified^d^	-	-	-	-	-
C12	40	5.5	unidentified^d^	-	-	-	-	-
C13	25	6.5	unidentified^d^	-	-	-	-	-
C11	40	5.5	0459	uncharacterized protein conserved in bacteria (PRC-barrel domain protein)	29,224	4.78	183	45
C1	70	5.5	1152	translation elongation factor G	75,079	4.73	1019	34

a , apparent mass and pI of the protein on 2D-IEF/SDS-PAGE.

b , molecular mass and pI as predicted by IMG's peptide statistics (PEPSTATS).

c , score and sequence coverage of the peptide fingerprint match as indicated by the MASCOT-search engine.

d , no significant match in all databases tested (see text).

Surprisingly, only few and only minor protein spots were observed exclusively in extracts of butyrate-grown *S. wolfei* cells (cf. Figs. 2A and 2B). The minor spot D5 validly identified Swol_2452, predicted to encode a zinc-containing carboanhydrase-like protein (COG0288). Spot D1 mapped Swol_2054, a ‘conserved hypothetical gene’, e.g. in *Clostridium* species, with putative domains for hydrolase activity (zinc-containing *beta*-lactamase fold, IPR001279), NADH-binding (IPR001327), FMN-binding (IPR008254), and rubredoxin-type iron binding (IPR004039). For the gel shown in Figure 2A, spots D2, D3 and D4 were inferred to result from protein degradation (e.g., by proteases), since they attributed a gene for a larger, constitutively expressed protein (spot E2, see below) and therefore were not listed in Table 2.

Most of the constitutively expressed soluble proteins in *S. wolfei* were attributed to genes for the butyrate oxidation pathway. The entry into the pathway, butyryl-CoA formation, was represented by protein spot E11 (Fig. 2AB) that identified Swol_1932, an acyl-CoA transferase/hydrolase gene (Table 2). Gene Swol_1932 clustered with other genes for short-chain acyl-CoA degradation (Swol_1933-36). The next gene in the cluster, Swol_1933, encoding the second enzyme in the pathway, butyryl-CoA dehydrogenase, was identified by a very prominent protein spot (E2 in Fig. 2AB). Swol_1933 had previously been identified to encode one of two butyryl-CoA dehydrogenases expressed in *S. wolfei* [14], and the second previously identified butyryl-CoA dehydrogenase, Swol_2052 [14], was matched by spot E4 (Fig. 2AB, Table 2).

For transfer of electrons from butyryl-CoA oxidation, two prominent spots, E17 and E13 (Fig. 2AB), attributed the two previously identified genes [14] for electron-transfer flavoproteins EtfA and EtfB, Swol_0697 and Swol_0696, respectively (Table 2). Notably, three sets of genes for EtfAB are encoded in the genome of *S. wolfei* [12], [14], however, the two identified ones are clustered with the expressed gene Swol_0698 (see above) for membrane-bound FeS-containing oxidoreductase protein (DUF224) that is attributed to act as EtfAB:quinone oxidoreductase (see Discussion). The crotonyl-CoA hydratase gene Swol_1936 in the gene cluster Swol_1933-36 was identified by the prominent spot E14 (Fig. 2AB), and the NAD-dependent 3-hydroxybutyryl-CoA dehydrogenase gene Swol_1935 by two prominent protein spots, E18 and E16 (Fig. 2AB). The previously identified NADH dehydrogenase (NDH) subunit encoded by Swol_1018 [14] was represented by spot E5, and is inferred to receive electrons *via* NADH from the 3-hydroxybutyryl-CoA dehydrogenase for further transfer to the hydrogenase/formate dehydrogenase subunits (see Discussion).

For *beta*-keto thiolysis of acetoacetyl-CoA, several protein spots mapped the same thiolase (acetyl-CoA acetyltransferase) gene, Swol_1934 in the Swol_1933-36 gene cluster, the major protein spots E8–E10 with identical molecular mass (appr. 45 kDa) but different isoelectric points (appr. pI 6.5–7 in Fig. 2), which is inferred to result from post-translational modifications of the proteins (e.g., decarboxylation, deamination). Swol_1934 encodes for a protein with predicted molecular mass of 41,283 Da and isoelectric point (pI) of pH 6.0 (Table 2). The sixth reaction step, from acetyl-CoA to acetyl phosphate, was represented by spot E12 (Fig. 2AB) which mapped a phosphotransacetylase gene, Swol_0767 (Table 2). The next gene in the genome, Swol_0768, encodes the last step in butyrate oxidation, the formation of ATP from acetylphosphate, and was represented by two prominent spots, E6 and E7, at the same molecular weight (appr. 45 kDa) but different pI (Fig. 2AB); also Swol_0768 had previously been identified to be expressed in *S. wolfei* [14]. Finally, spots E1 and E3 attributed ATP-synthase subunit genes (see above) and spot E15 a gene for vitamin B6 biosynthesis (Table 2).

The prominent spots that appeared exclusively in extracts of crotonate-grown cells, i.e., the crotonate-inducible proteins, were also excised and submitted to PF-MS. One set of spots that appeared uniquely on gels of crotonate-grown cells, C2–C4 at around 60 kDa molecular mass (cf. Figs. 2B and 2A), validly identified two different [FeFe] hydrogenase catalytic subunit genes (Table 2). Spot C2 and C3 identified Swol_1017, which we previously identified [14] to contribute one component to a membrane-associated, internally oriented NADH:hydrogenase/formate-dehydrogenase complex in butyrate-grown *S. wolfei* (see Discussion) (HYD-1; [12]). However, spot C4 (as well as peptides of spot C3) identified Swol_2436, which is predicted to encode yet another [FeFe] hydrogenase large subunit (HYD-3). The two genes, the previously identified Swol_1017 and the newly identified Swol_2436, share 68% sequence identity at the amino acid level. Notably, spot C4 seemed to be more prominent than spot C2; thus, it can be inferred that in the crotonate-grown cells that we analyzed, most of the catalytic component of a soluble hydrogenase complex was expressed from gene Swol_2436 rather than from Swol_1017.

Other prominent protein spots visible uniquely on gels from crotonate-grown cells, spot C5 and C14, identified Swol_0436 and the next gene in the genome, Swol_0435, annotated as acetyl-CoA hydrolase/transferase and 3-hydroxybutyryl-CoA dehydrogenase, respectively. Hence, during growth with crotonate, both genes seemed to be expressed in addition to the readily identified, iso-functional, and constitutively expressed genes Swol_1932 and Swol_1935 (see above). Similarly, spot C7 identified Swol_2126 for another short-chain specific acyl-CoA dehydrogenase, spot C15 identified Swol_2030 for another 3-hydroxyacyl-CoA dehydrogenase, and spot C10 identified Swol_0675 for another acetyl-CoA acetyltransferase.

Surprisingly, for the crotonate-inducible spots C8, C9, C12 and C13, we were not able to validly match the obtained peptide fingerprints to any predicted gene in the annotated *S. wolfei* genome sequence in several attempts, and not when searching against the nucleotide sequence (six frames) of *S. wolfei*, or against the NCBI GenBank; hence, their identity and origin remain unclear. Finally, spot C11 identified Swol_0459 predicted to encode a hypothetical protein containing a PRC-barrel domain (IPR007903), spot C6 identified Swol_0412 as predicted [4Fe4S]-subunit gene of 2-hydroxyacyl-CoA dehydratase/benzoyl-CoA reductases, and spot C1 identified a predicted translation-elongation factor gene (see Table 2).

### Proteins identified by formate dehydrogenase and hydrogenase-activity staining and peptide fingerprinting-mass spectrometry

**Figure 3:**
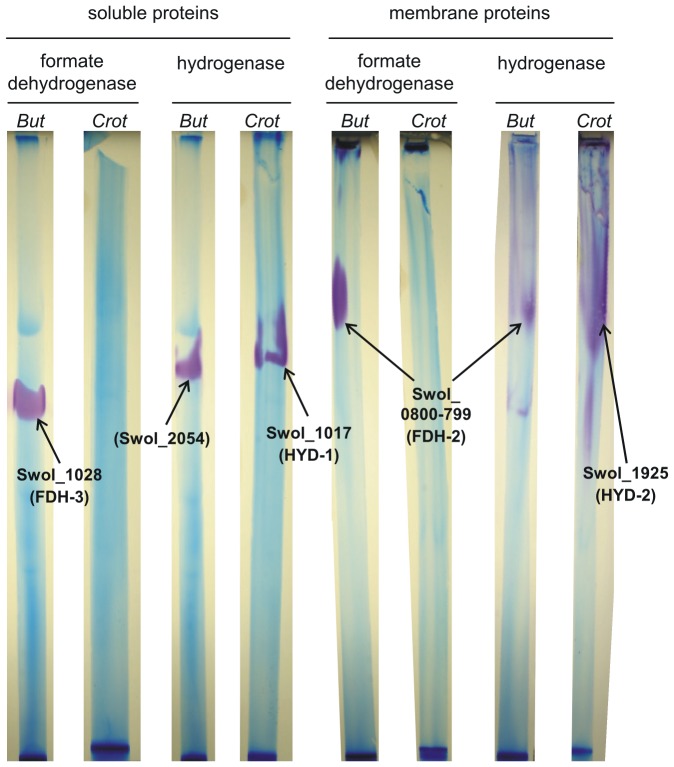
Blue-Native PAGE gel strips after formate dehydrogenase or hydrogenase activity staining. Soluble or membrane proteins of butyrate- or crotonate-grown *S. wolfei* were separated by Blue-Native PAGE under anoxic conditions, and the individual lanes excised from the gels and submitted to formate dehydrogenase or hydrogenase activity staining when using formate or hydrogen gas as the electron donor, respectively, and the electron acceptor benzyl viologen as the stain. Stained bands were excised and analyzed by PF-MS in order to identify the corresponding formate dehydrogenase or hydrogenase catalytic subunits. The figure illustrates the endpoint of the staining and the formate dehydrogenase or hydrogenase catalytic subunits identified (locus tags and numbering of the catalytic subunits according to Sieber *et al.* [12]); for images showing the time course of the staining, and for details on the PF-MS identifications, see Figs. S3, S4, S5, S6 and Table S1 in the Supplemental information file, respectively. Legend: *But* and *Crot*, soluble or membrane proteins from butyrate- and crotonate-grown *S. wolfei* cells.

The results described above were confirmed, and expanded upon, when we established anoxic Blue-Native PAGE and activity staining of protein bands (see Material and Methods) in order to separate and identify formate dehydrogenase and hydrogenase activities in the soluble and membrane fractions of butyrate- and crotonate-grown *S. wolfei* cells (Fig. 3; for details, see Tables S1 and S2, and Figs. S3, S4, S5, S6, and S7 in the Supplemental information).

Most importantly, formate dehydrogenase activity could be detected only in butyrate-grown cells, but not in crotonate-grown cells: as illustrated in Figure 3, PF-MS of a stained, very prominent band that indicated a membrane-associated formate dehydrogenase in butyrate-grown cells re-identified Swol_0800-799, hence, the FDH-2 catalytic subunit (see above) (see also Fig. S3 and Table S1). For the experiment shown in Figure 3, PF-MS of a band that indicated another formate dehydrogenase activity in the soluble fraction of butyrate-grown cells (Fig. 3; see also Fig. S4 and Table S1) identified yet another FDH catalytic subunit, Swol_1028 (Fig. 3; see also Table S1), of the FDH-3 complex [12]. Interestingly, in an independent experiment with crude extract prepared from a different batch of butyrate-grown *S. wolfei* cells (see Fig. S7 and Table S2), the stained band attributed the previously identified FDH-1 catalytic subunit genes, Swol_0785–86 (selenocysteine-linked FDH-1 subunit) [14] as well as the previously identified HYD-1 hydrogenase catalytic subunit, Swol_1017 [14].

In contrast, hydrogenase activities were detectable in both, butyrate- and crotonate-grown cells, and in both, membrane and soluble fractions, respectively (Fig. 3 and Figs. S5, S5, S6): PF-MS of the strongly stained, prominent bands that indicated soluble hydrogenase in crotonate-grown cells each re-identified Swol_1017, i.e., the HYD-1 catalytic subunit (see above) (Table S1). On the other hand, PF-MS of a faint band that indicated a membrane-associated hydrogenase activity in butyrate-grown cells identified again Swol_0800-799, hence, the FDH-2 catalytic subunit. Furthermore, a broad but weakly stained band indicative of a membrane-associated hydrogenase activity in crotonate-grown cells (see Fig. 3) attributed yet another hydrogenase catalytic subunit, Swol_1925 (Fig. 3, Fig. S5 and Table S1) of the HYD-2 complex [12]. Finally, PF-MS of a band that indicated a soluble hydrogenase activity in butyrate-grown cells delivered no valid identification of a hydrogenase (or formate dehydrogenase) catalytic subunit gene (Fig. 3, Fig. S6 and Table S1); interestingly, the only protein with (predicted) oxidoreductase/electron carrier activity that could be identified for this band was ‘putative flavoprotein’ Swol_2054, i.e., the butyrate-inducible, soluble protein that was identified with spot D1 (see Fig. 2A and Table 2) and that contains conserved domains for zinc-binding, NADH-binding, FMN-binding, and rubredoxin-type iron binding (see above).

### Activity and localization of formate dehydrogenases and hydrogenases in intact cells and cell extracts

The results documented above were examined by measurement of formate dehydrogenase and hydrogenase activities detectable in suspensions of intact cells and in cell extracts, when assayed in reverse as benzyl viologen (or NAD^+^) reducing activity (Table 3).

**Table 3:** Formate dehydrogenase and hydrogenase activities detectable in intact cell suspension, in crude extract, and in soluble fraction of butyrate and crotonate-grown cells of *S. wolfei*.

Enzyme activity	Formate dehydrogenase (mU/g protein^b^)			Hydrogenase (mU/mg protein^b^)		
Growth substrate	Butyrate		Crotonate	Butyrate		Crotonate
**Electron acceptor**	BV	NAD^+^	BV	BV	NAD^+^	BV
**Cell suspension**	894.4±149.6	13.3±1.4	2.2±0.1	205.5±70.7	2.6±0.9	703.2±189.7
**Cell-free supernatant** a	60.6±18.3	n. d.	0.2±0.3	29.5±2.5	n. d.	37.4±8.3
**Crude extract**	484.2±283.9	54.8±26.0	2.0±0.9	720.5±123.4	0.7±0.1	842.9±136.2
**Soluble fraction**	129.5±29.8	127.7±16.4	n. d.	32.1±11.6	2.0±0.3	n. d.

a , activities in the supernatant of cell suspensions were determined in order to confirm that the cells had remained intact.

b , specific activities refer to protein concentration of crude extract.

n. d., not determined.

BV, benzyl viologen.

Intact cells of syntrophically butyrate-grown cells exhibited a very high benzyl viologen (BV)-dependent formate dehydrogenase activity (but not with NAD^+^), whereas no significant activity was detectable with crotonate-grown cells (Table 3). Hence, the active centre of this formate dehydrogenase must be oriented externally to the cytoplasmic membrane, since the cytoplasmic membrane is not permeable to BV [16]. Disruption of cells and of cytoplasmic membranes through French-Press treatment decreased the BV-reducing formate dehydrogenase activity by nearly 50% (see Table 3, crude extract), and the remaining activity was mainly associated to the membrane (Table 3, soluble fraction). However, the cell extract exhibited also significant NAD^+^-reducing formate dehydrogenase activity (Table 3, crude extract), and this activity was localized predominantly in the cytoplasmic fraction (Table 3, soluble fraction). Notably, none of these formate dehydrogenase activities was detectable in crotonate-grown cells at significant levels.

Suspensions of intact butyrate-grown cells exhibited also BV-dependent hydrogenase activity, but this activity was much higher in crotonate-grown cells (Table 3). This hydrogenase activity was nearly exclusively membrane-associated and showed very little activity with NAD^+^ as electron acceptor. However, a major NAD^+^-dependent hydrogenase activity was found in the cytoplasmic fraction of crotonate-grown cells, as well as in the cytoplasmic fraction of butyrate-grown cells (Table 3).

Finally, suspensions of intact, butyrate-grown cells exhibited significant BV-reducing activity also in the absence of formate or hydrogen as electron donor (Table 4), and this endogenous activity was strongly inhibited by addition of the protonophore CCCP, hence, was suggested to be linked to a functional proton gradient in intact cells. Importantly, when the BV-reducing activity had been stimulated by addition of formate or hydrogen as electron donor, also the BV-reducing formate dehydrogenase activity was strongly inhibited by CCCP whereas the BV-reducing hydrogenase activity was inhibited to a lesser extent (Table 4).

**Table 4:** Inhibition of benzyl viologen-reducing activities detectable in intact cell suspensions of butyrate-grown *S. wolfei* after the addition of protonophore CCCP (see also text).

Enzyme activity (mU/mg protein)	No CCCP	With CCCP	Relative inhibition (%)
BV-reducing activity^a^	225.6±48.8	49.2±3.1	78
Formate-dependent BV-reducing activity	973.0±112.7	438.3±39.9	55
Hydrogen-dependent BV-reducing activity	246.9±45.5	172.6±80.3	30

a endogenous BV-reducing activity of whole cells in the absence of formate or hydrogen as external electron donor.

BV, benzyl viologen.

## Discussion

**Figure 4:**
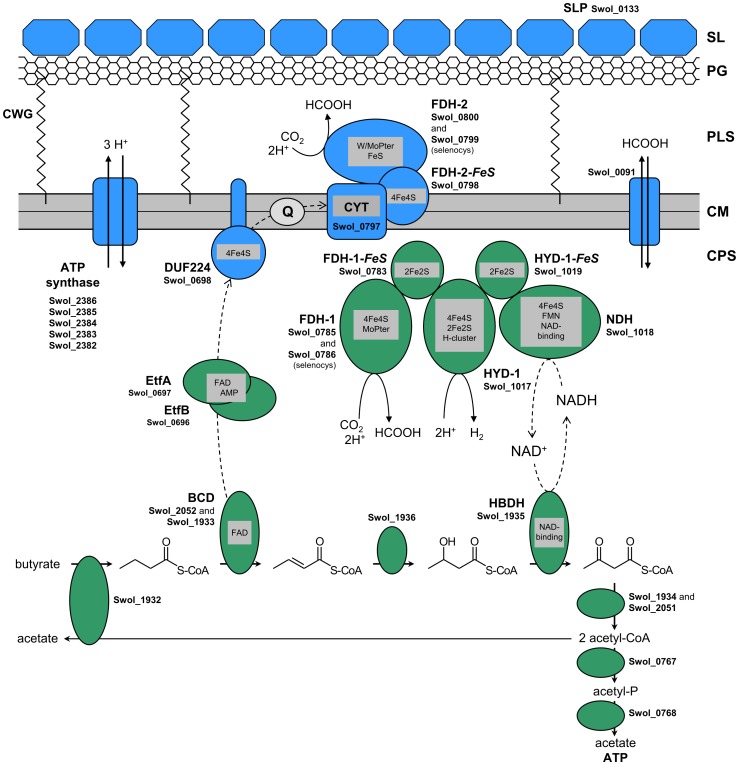
Schematic representation of the proteins identified in syntrophically butyrate-grown *S. wolfei* cells. The illustration includes the proteins attributed to butyrate oxidation, electron flow, formate or hydrogen formation, ATP conversion, and cell-wall structure as identified in the present and in our previous study [14], and their attributed location in *S. wolfei* cells. Proteins colorized in green were identified in the soluble fraction, and proteins colorized in blue in the membrane fraction, of syntrophically butyrate-grown *S. wolfei* cells. Dashed arrows indicate the flow of electrons. Legend: FDH, formate dehydrogenase; HYD, hydrogenase; BCD, butyryl-CoA dehydrogenase; EtfA and EtfB, electron transport flavoproteins A and B; DUF224, membrane-bound FeS-containing oxidoreductase; CYT, membrane-bound cytochrome; Q, quinone; HBDH, 3-hydroxybutyryl-CoA dehydrogenase; NDH, NADH dehydrogenase; SLP, surface-layer (S-layer) protein; CWG, cell-wall glycopolymers; SL, surface layer; PG, peptidoglycane; PLS, periplasma-like space; CM, cytoplasmic membrane; CPS, cytoplasmic space.

Resolving the mystery of how *Syntrophomonas wolfei* couples fermentation of butyrate to acetate with hydrogen/formate formation, which is energetically unfavourable, is a particularly difficult challenge [17]. A very essential step forward was the sequencing and thorough annotation of the genome of *S. wolfei* [12]. Another recent and important step was the purification and identification of specific enzymes of *S. wolfei*, e.g., an NADH:acceptor oxidoreductase activity and butyryl-CoA dehydrogenase activity [14]. In the present study, all proteins in *S. wolfei* that are highly expressed during syntrophic growth with butyrate were compared by protein gel electrophoresis with those expressed during pure culture growth with crotonate, and identified by peptide fingerprinting-mass spectrometry (PF-MS). The rationale for our gel-based proteomic approach was that all highly abundant proteins visible on gels can be considered to be important for some aspects of cellular function, especially in an organism such as *S. wolfei* that grows under such a difficult energetic condition and therefore has to economize its energy consumption, e.g., in protein synthesis. Furthermore, our gel-based proteomic approach allowed to evaluate not only the relative abundance of proteins (by their band intensities), but also the PF-MS identifications obtained when comparing the observed molecular masses of the proteins (and their isoelectric points in case of 2D-gels) with the predicted molecular masses (and pI) derived of the respective attributed gene sequences. The results obtained (Figs. 1AB, 2AB, S1, S2 and Tables 1 and 2) in combination with results from Blue-Native PAGE and activity staining (Figs. 3, S3, S4, S5, S6, and S7 and Table S1 and S2) and enzyme measurements in intact cells and cell extracts (Tables 3 and 4) allow to derive a first evidence-based concept of the electron flow and energy economy for this unusual type of metabolism operating close to the minimum energy yield for microbial growth (Fig. 4).

According to the reaction schema depicted in Figure 4, butyrate is activated by a CoA-transferase exchanging with acetyl-CoA to form butyryl-CoA. *Beta*-oxidation proceeds *via* crotonyl-CoA, 3-hydroxybutyryl-CoA, and acetoacetyl-CoA, to form two acetyl-CoA, one of which exchanges CoA with butyrate, and the other one yields one ATP *via* the phosphotransacetylase and acetate kinase reactions [8]. Proteins representative for all these reaction steps could be identified in the soluble protein fraction (Fig. 2A, Table 2), with butyryl-CoA dehydrogenase (BCD) (spot E2 in Fig. 2A), 3-hydroxybutyryl-CoA dehydrogenase (spot E18), and acetyl-CoA acetyltransferase (spots E8-10) being among the most abundant proteins detectable in the soluble fraction. These proteins were expressed from a single gene cluster for short-chain acyl-CoA degradation, the previously identified Swol_1932-36 gene cluster [14]. Notably, the same gene cluster was obviously expressed during pure culture growth of *S. wolfei* with crotonate, however, it appeared that also several iso-enzymes were expressed in these cells (discussed further below).

In the 3-hydroxybutyryl-CoA dehydrogenase reaction, electrons are transferred to NAD^+^ to form NADH. The NADH can then be used to reduce either protons to molecular hydrogen, or CO_2_ to formate (Fig. 4). The redox potential of the 3-hydroxybutyryl-CoA dehydrogenase reaction (-250 mV) is sufficiently low to allow the release of these electrons *via* NADH to hydrogen at 10^−4^–10^−5^ atm, corresponding to the conditions measured in fatty acid-oxidizing co-cultures or in methanogenic bioreactors [1]. In analogy, CO_2_ reduction to formate under our cultivation conditions, i.e., with a background CO_2_ concentration of 10^−1^ atm, would require to maintain formate concentrations in the range of 1–10 µM. The expression of the protein complex that was identified and interpreted previously to likely represent a membrane-associated, internally oriented NADH:hydrogenase/formate-dehydrogenase complex [14] (NDH/HYD-1/FDH-1 complex, see Fig. 4) could be confirmed in this study, but the parent proteomic analysis yielded a more refined picture with regard to the abundance of the individual components of the complex. The NADH:acceptor oxidoreductase (NDH) component (Swol_1018) was always present as a prominent protein spot visible at about the same intensity in the soluble fractions of both, butyrate- and crotonate-grown cells (spot E2, cf. Figs. 2A and 2B). On the other hand, the HYD-1 catalytic component (Swol_1017) and the FDH-1 catalytic component (Swol_0785–86, selenocysteine-linked) could never be detected among the highly abundant proteins in the soluble or membrane fraction of butyrate-grown cells (in contrast to crotonate-grown cells, where the HYD-1 component was always highly abundant [cf. spots C2-C4 in Fig. 2B]). Nevertheless, the latter two components, HYD-1 and FDH-1, could be co-identified in butyrate-grown cells when we used activity staining with high sensitivity to detect the catalytic subunits after separation by Blue-Native PAGE (Fig. S7 and Table S2; band CE3). Hence, the HYD-1 and FDH-1 components in butyrate-grown cells are likely expressed only at basal levels in butyrate-grown cells, and thus are not visible as bands on gels unless co-purified with the NADH:acceptor oxidoreductase activity (see ref. 14). Furthermore, it appeared that, depending on the batch of *S. wolfei* cells used, the FDH-1 component could be replaced by the FDH-3 catalytic component as illustrated for the batch of cells used for the experiment shown in Figure 3 (and in Fig. S4 of the Supplemental information); here, the activity staining and PF-MS identified the FDH-3 subunit Swol_1028 instead of the selenocystein-linked FDH-1 subunit Swol_0785-86 (cf. Tables S1 and S2).

The electrons released in the butyryl-CoA dehydrogenase reaction (E_0_′ = −125 mV, ref. 7; E_0_′ = −10 mV, ref. 10) are transferred *via* a flavin-containing electron transfer component EtfAB to the electron carrier protein DUF224 which is anchored in the cytoplasmic membrane (Fig. 4). The EtfAB proteins were among the most abundant proteins detectable in the soluble fraction (spots E13, E17 in Fig. 2AB), and the DUF224 protein was abundant in the membrane fraction (band A7 in Fig. 1A), both after growth with either butyrate or crotonate; the coding genes are co-expressed from a single gene cluster (Swol_0996-98) attributed previously [14]. The DUF224 is anticipated to contain a menaquinone-binding site to transfer electrons to menaquinone (E_0_′ = −74 mV) [12] and the presence of menaquinone in *S. wolfei* has been documented earlier [11]. Hence, the four proteins likely constitute a membrane-associated BCD/EtfAB/DUF224 complex which transfers electrons from the crotonyl-CoA/butyryl-CoA redox couple into the menaquinone pool of the membrane (Fig. 4). Notably, the EtfAB expressed in *S. wolfei* seems to be more closely related to the group I (‘aerobic’-type) of EtfABs than to the group II (‘anaerobic’ or ‘bifurcating’-type) of EtfABs [18] (see the phylogenetic tree in the Supplemental information, Fig. S8).

From the menaquinone cycle, electrons can be transported through a membrane-bound cytochrome (CYT in Fig. 4) that couples *via* a 4Fe4S-containing electron carrier (FDH-2-*FeS*) directly to an externally oriented formate dehydrogenase (FDH-2). The FDH-2 enzyme complex was consistently found to be expressed at very high levels only in syntrophically butyrate-grown cells but not in crotonate-grown cells (Figs. 1, 3, S1, S2 and Tables 1, 3 and S1), and would use the electrons from butyryl-CoA oxidation for CO_2_ reduction to formate. Thus, formate would be the preferential electron carrier in syntrophic butyrate oxidation by *S. wolfei*. Formate as an alternative electron carrier in syntrophic butyrate oxidation has been discussed since long [1], [17] because the partner organisms coupling with *S. wolfei* can use both, hydrogen and formate, as electron donors. Alternatively, formate could pass the cytoplasmic membrane *via* the identified formate transporter (Swol_0091 in Fig. 4) [19] and could be exchanged into hydrogen as electron carrier at the hydrogenase/formate dehydrogenase (HYD-1/FDH-1) complex. Thus, *S. wolfei* can use either formate or hydrogen as electron carrier to the methanogenic partner, and may even use both simultaneously, just depending on the concentrations of the respective carriers outside of the cell. Interspecies electron transfer *via* formate appears to play an essential role also in syntrophic propionate oxidation by *Syntrophobacter fumaroxidans* [20], together with interspecies hydrogen transfer. In this bacterium, also an Rnf complex (i.e., a proton-translocating ferredoxin:NAD^+^ oxidoreductase) appears to contribute significantly to energy conservation [20], and a Rnf complex was also found in *Syntrophus aciditrophicus* and may serve an important function in fatty acid oxidation also in this bacterium [2], [3]. However, in *S. wolfei*, an Rnf complex is missing and there is no oxidation step involved in butyrate oxidation that can be coupled directly to ferredoxin reduction [12], [14].

Formate production outside of the cell with protons pumped actively from the inside to the outside of the cell, is an elegant strategy to bridge the redox potential difference between the butyryl-CoA dehydrogenase reaction and formate formation. Through the reaction chain described, protons are released on the inside of the cell, at the latest in the reaction of menaquinol with the cytochrome, however, the consumption of protons outside of the cell during formate formation is enforced through the proton potential across the membrane. If we assume a proton-motive force across the cytoplasmatic membrane in a similar range as in other bacteria, i.e., about −180 mV, this potential could easily drive extracellular formate formation, bridging the redox potential difference between −74 mV and −250 to −300 mV. Whether the menaquinone cycle would also be driven by this proton potential is so far still open. However, our experiments with intact cells (Table 4) and earlier results [11] with and without the protonophore CCCP, strongly support the notion that a proton potential across the cytoplasmatic membrane is essential in the overall process. The proton potential would be maintained by ATP hydrolysis through the F_1_F_O_ ATPase (Fig. 4); notably, the ATPase of *S. wolfei* most likely pumps protons rather than sodium ions based on the amino-acid sequence of its c-subunit [21] (see sequence alignment in the Supplemental information, Fig. S9), however, this cannot be predicted unambiguously by sequence comparison alone [22].

In summary, the suggested model of reversed electron transport is a reversal of the concept of electron transport phosphorylation assumed to fuel the energy metabolism of *Wolinella succinogenes* [23], [24], which has previously been suggested to drive also the endergonic oxidation of succinate during syntrophic oxidation of propionate in *Syntrophobacter fumaroxidans* [3], [20]. With this, the overall energy balance of *S. wolfei* could be closed as follows: if the ATPase transports three protons per ATP across the membrane, the elevated proton concentration outside of the cell in comparison to inside could be sufficient to allow CO_2_ reduction with two protons and the two electrons from butyryl-CoA oxidation, and the equivalent of one proton, i.e., one-third of an ATP equivalent or roughly −20 kJ per mol butyrate oxidized, would remain to drive all biosynthetic processes for cellular biomass formation. This energy value had been calculated to be available to syntrophic butyrate oxidizers in natural or close-to-nature conditions [1] and is close to the minimum amount of energy needed for microbial life at all. If the stoichiometry of the ATPase is four rather than three protons per ATP hydrolysed, the overall energetics are shifted slightly to the better towards a higher remnant energy yield for growth.

Interestingly, the most abundant protein detectable in the membrane fraction of *S. wolfei* cells grown with either butyrate or crotonate, was represented by ‘hypothetical outer-membrane protein’ Swol_0133, which we predict to represent a surface-layer (S-layer) protein (Fig. 4). S-layer (glyco)proteins can make up 10–20% of the total cellular protein (see band B2 in Figs. 1A and S2), exhibit low sequence homology (Swol_0133 shows only 27% sequence identity to characterized S-layer glycoproteins, e.g., to SatA of *Aneurinibacillus thermoaerophilus*), and form a regularly ordered, planar array of subunits yielding a complete shielding of the bacterial cell, thereby generating a functional equivalent to the periplasm of Gram-negative bacteria [15], [25]–[27]. Importantly, for *S. wolfei* that phylogenetically belongs to the phylum Firmicutes [12], [28], such a multi-layered cell wall including a ‘periplasm-like structure’ has been observed previously [29]. Furthermore, the cell wall of other Firmicutes, e.g., of *Bacillus subtilis* and *Staphylococcus aureus*, has been shown to act like a cation exchanger that retains protons from the respiratory metabolism within the cell wall, e.g., bound to glycopolymers [30]–[32], and one of the biological functions discussed also for the S-layer is binding of protons in order to maintain an acidic cell wall [33] due to its weakly acidic nature at neutral pH (the pI predicted for Swol_0133 is pH 4.7). We therefore suggest that the cell wall of *S. wolfei* and the macromolecules contained therein (e.g., S-layer proteins, glycopolymers, peptidoglycan, and/or phospholipid-layer) may help to maintain a locally increased proton concentration and promote formate formation outside the cytoplasmic membrane (Fig. 4) by preventing a ‘futile escape’ of protons into the bulk medium. These macromolecules might even directly constitute a ‘localized proton-relay pathway’ (see ref. [34] and the refs. cited therein) in order to effectively feed protons from the ATPase complex into the formate dehydrogenase complex in *S. wolfei*.

Finally, the proteomic analyses confirmed furthermore that the *S. wolfei* variant that is able to grow axenically with crotonate [35] differs significantly in its metabolic features from syntrophically butyrate-grown cells [9]. Besides the differences in the hydrogenase and formate dehydrogenase expression pattern discussed above, crotonate-utilizing cells appeared to express several iso-enzymes for fatty acid degradation compared to butyrate-utilizing cells (Table 2, Fig. 2), for example, an additional CoA-transferase-like protein (Swol_0436; prominent spot C5 in Fig. 2B) that could represent the crotonyl-CoA:acetyl-CoA transferase activity detected previously specifically in crotonate-grown cells [9], and an additional NAD^+^-dependent 3-hydroxybutyryl-CoA dehydrogenase (Swol_0435; prominent spot C14 in Fig. 2B) that might be involved in a more efficient NAD^+^-regeneration in cells disproportionating crotonate to butyrate and acetate [9]. Notably, four additional prominent spots that appeared solely for crotonate-grown cells (spots C8, C9, C12 and C13 in Fig. 2B) could not be identified in this study (the proteins produced high-quality peptide fingerprints, but no valid matches could be found in the databases). Hence, their origin, identity, and their function in crotonate-grown cells, remains also to be clarified in future studies.

## Materials and Methods

### Organisms, growth media and incubation

*Syntrophomonas wolfei* subsp. *wolfei* [29], [36] in co-culture with *Methanospirillum hungatei* JF1 (DSM 2245B) was grown in anoxic, bicarbonate-buffered and sulfide-reduced freshwater medium [37], [38] containing 0.05% yeast extract, 0.4 mg/L resazurine as redox indicator, EDTA, a decreased amount of iron to minimize precipitation of iron sulfide [39], and 20 mM sodium butyrate; the 7-vitamin solution of the original freshwater medium was supplemented with lipoic acid (200 µg/L) and thiamine (400 µg/L) as described earlier [40]. Axenic cultures of *S. wolfei* were grown with 20 mM sodium crotonate [11]. The media were prepared in 4-L jars and distributed to 1-L or 120-mL infusion bottles after autoclaving for 40 min as described earlier [39], or directly in 10-L culture vessels. Cultures were incubated at 30°C in the dark under N_2_/CO_2_ (80∶20) atmosphere. Growth was monitored *via* optical density (OD 580 nm) against sterile medium; a few grains of sodium dithionite were added to the cuvettes to keep resazurine in its reduced state. Anoxic buffers for cell harvest or cell suspension experiments were prepared as described previously [14].

### Harvesting and preparation of cell suspensions

Cultures were harvested at the end of the exponential growth phase (OD_578_ = 0.1–0.18 after approximately 10–20 days) in an anoxic chamber (Coy, Ann Arbor, USA) by centrifugation as described earlier [14]. Cells were washed twice by repeated centrifugation in anoxic 50 mM potassium phosphate buffer, pH 7.5, and resuspended in 4–6 mL of the same buffer. Cells of *S. wolfei* and *M. hungatei* were separated as described earlier [14] *via* a Percoll gradient from 55 to 70% [41]. Briefly, after centrifugation (2,200×g, 1 h), the upper, *S. wolfei*-containing layer was transferred to infusion bottles and washed twice in anoxic buffer by repeated centrifugation (2,600×g, 20 min); the cell pellet was suspended in 5 mL of 20 mM Tris-HCl, pH 8.0, for cell lysis, or in 3 mL 50 mM potassium phosphate buffer, pH 7.5, with 3 mM DTT for cell suspension experiments (see below).

### Preparation of cell extracts and subcellular fractionation

Cells were opened by three passages through an anoxic, cold French Pressure cell operated at 137 MPa. The cell lysate was collected in an 8 mL serum vial and the cell debris removed by centrifugation in an SS-34 rotor at 3,000×g for 20 min. The supernatant obtained (cell-free extract) was further fractionated in an Optima TL-ultracentrifuge using the TLA-100.4-rotor (Beckman) at 236,000× *g* for 30 min, which yielded the soluble protein fraction (supernatant) and the membrane fraction (pellet).

### Solubilisation of membrane proteins

The membrane pellet obtained from ultracentrifugation was resuspended in 4 mL anoxic potassium phosphate buffer and washed twice in anoxic potassium phosphate buffer by repeated resuspension and centrifugation at 236,000×g for 30 min. The washed membrane particles were carefully resuspended in 4 mL of 20 mM Tris-HCl, pH 8.0, containing 0.5% dodecyl β-D-maltoside. After incubation on ice for 30 min, this mixture was centrifuged (236,000×g, 30 min) and the obtained supernatant was termed solubilised membrane proteins.

### Activity measurements

For measurement of formate dehydrogenase and hydrogenase and determination of their localizations in *S. wolfei*, enzyme assays with intact cells, crude extract, and soluble protein extract were run anoxically in rubber-stoppered 1-mL cuvettes at 30°C as described earlier [39]; assays were run in triplicate. Activity was expressed in units (U) defined as 1 µmol substrate consumed/product formed per min; specific activity was expressed as U/mg total protein. Hydrogenase (EC 1.18.99.1) was assayed in 50 mM potassium phosphate buffer, pH 7.5, with 3 mM DTT in cuvettes flushed with 100% H_2_ gas. As electron acceptor, 2 mM benzyl viologen (ε_578_ = 8.65 mM^−1^ cm^−1^ [42]) or 0.25 mM NAD^+^ (ε_340_ = 3.5 mM^−1^ cm^−1^ [43]) was used; cell extract was added and the increase of absorption was followed at 578 nm or 340 nm, respectively (modified from ref. [44]). Formate dehydrogenase (EC 1.2.1.2) was assayed under the same conditions as hydrogenase (except H_2_ gas) and the reaction started by addition of 5 mM formate. In assays testing the effect of the protonophor carbonyl cyanide *m*-chlorophenyl hydrazone (CCCP) on formate dehydrogenase and hydrogenase activity, cell suspensions were pre-incubated anoxically at 30°C for 15 min in the presence or absence of 15 µM CCCP.

### Chemicals

All standard chemicals were of analytical or higher grade quality and obtained from Boehringer, Eastman Kodak, Merck, Pharmacia, Serva, or Sigma. Gases were purchased from Messer-Griesheim (Darmstadt, Germany) and Sauerstoffwerke Friedrichshafen (Friedrichshafen, Germany).

### Analytical methods

Protein concentration was determined by the Bradford microprotein assay [45] using bovine serum albumin as standard.

### Protein electrophoresis

One-dimensional SDS-PAGE was done according to Laemmli [46]. Gels contained either 12% polyacrylamide in the resolving gel and 4% polyacrylamide in the stacking gel, or gradients gels 5–18% polyacrylamide in the resolving gel and 4% polyacrylamide in the stacking gel, and cast as large gels (17 cm by 20 cm, Protean II xi, BioRad) for the excision of bands to be analyzed by peptide fingerprinting-mass spectrometry (see below). Protein samples were mixed 1∶2 with loading buffer (0.125 M Tris-HCl, pH 6.8, 2% (w/v) SDS, 25% glycerol, 0.01% (w/v) bromophenolblue and 5% *β*-mercaptoethanol) and heated at 100°C for 5 min prior to loading. Protein separation was started with 15 mA, and after the marker front had reached the resolving gel the current was increased to 25 mA; the gel-chamber was cooled to 8°C during the runs. Gels were stained with colloidal Coomassie blue [47].

Two-dimensional isoelectric-focussing (IEF)/SDS-PAGE was done using the BioRad ReadyStrip IPG system for the first-dimension separation (17 cm length, pI range pH 3–10 or pH 5–8), and for the second-dimension SDS-PAGE as described above (17 cm by 20 cm; 12% polyacrylamid, no stacking gel). The sample preparation and IEF-separation conditions were essentially as described in the manufacture's instructions (BioRad's ReadyStrip IPG Strip Instruction Manual) with the following modifications. Soluble proteins obtained after ultracentrifugation of crude extract (see above) were desalted using PD-10 desalting columns (GE Healthcare Life Sciences). One-mg aliquots of desalted soluble protein were precipitated by addition of four volumes of ice-cold acetone; the suspension was stored at −20°C overnight, and the protein collected by centrifugation (16,000× *g*, 15 min, 4°C). The protein pellet was dried under air (appr. 15 min) and solubilized in 300 µL rehydration buffer as described in the ReadyStrip IPG Strip Instruction Manual. The IPG strips were rehydrated with the protein sample (in 300 µL) overnight. The isolelectric-focussing conditions involved a maximal current of 50 µA per strip at 20°C and started for 1 h with a maximal voltage of 500 V (desalting), followed by a voltage ramp (rapid) to a maximal voltage of 10,000 V during 3 h, and additional focusing at 10,000 V until a total of 40,000 Volt-hours (Vh) had been reached. Thereafter, each strip was equilibrated in SDS-equilibration buffers I and II (with DTT and iodoacetamide, respectively) as described in the ReadyStrip IPG Strip Instruction Manual, and each equilibrated strip placed onto one SDS-PAGE gel (see above) using an overlay of Tris-Glycin-SDS buffer solidified with agarose (0.5%).

Two-dimensional Blue-Native PAGE/SDS-PAGE for the separation of protein complex components from membranes was done following the protocol of Wittig *et al.* [48]. Briefly, a Blue-Native gel with 4–13% polyacrylamide gradient was prepared (17 cm by 20 cm). Samples (50 µL) containing appr. 200 µg total protein from dodecylmaltoside-solubilized membrane fragments (see above) were treated prior to loading onto the Blue-Native gel by addition of Coomassie blue G-250 (1 µL of 5% w/v) and glycerol (5 µl of 50% v/v). Treated samples were loaded and separated at 10 mA for 4 h. For a second-dimension separation *via* denaturing SDS-PAGE, each lane of first-dimension separated membrane proteins of butyrate-grown *versus* crotonate-grown *S. wolfei* cells (see Results) was excised from the Blue-Native gel (as a slice of appr. 1 cm by 7 cm), equilibrated for 30 min in Tris-Glycin-SDS buffer containing additional SDS (1% w/v) and *β*-mercaptoethanol (0.5% v/v), and washed twice in Tris-Glycin-SDS buffer. Both equilibrated gel slices were placed onto one SDS-PAGE gradient gel (5–18% polyacrylamide, see above) using an overlay of Tris-Glycin-SDS buffer solidified with agarose (0.5%).

Anoxic Blue-Native PAGE for activity staining was done as described above with the following modifications. The cell extracts were processed anoxically. The membrane fragments were washed once and solubilised with dodecylmaltoside (see above). A total of 500 µg of protein per sample was separated (6 h at 400 V) on a gradient gel (4–13% polyacrylamide) in an anoxic chamber. Each lane was cut (as a slice of appr. 1 cm by 18 cm) and washed in anoxic 50 mM potassium phosphate buffer, pH 7.5, and placed into an anoxic staining box. After 10 min of purging with nitrogen gas, 25 mL anoxic potassium phosphate buffer (50 mM, pH 7.5) containing 1 mM benzyl viologen was injected per gel slice. Activity staining was started by addition of 5 mM formate or H_2_ gas. Stained bands were excised and submitted to peptide fingerprinting-mass spectrometry (see below).

### Peptide fingerprinting-mass spectrometry and database searching

Protein bands (or spots) of interest were excised from gels and submitted to peptide-fingerprinting-mass spectrometry at the Proteomics Facility of the University of Konstanz (www.proteomics-facility.uni-konstanz.de) to identify the corresponding genes. The MASCOT engine (Matrix Science, London, UK) [49] was used to match each peptide fingerprint against a local database of, firstly, all predicted protein sequences of the annotated genomes of *Syntrophomonas wolfei* subsp. *wolfei* strain Göttingen (GOLD Project Id: Gc00427; IMG version 2011-08-16). If these searches gave no valid matches (see Results) or for control, the fingerprint was matched against a database of the nucleotide sequence of the *S. wolfei* genome translated on all six reading frames, in order to identify also genes that could have been missed by primary ORF-calling and auto-annotation; the fingerprints were also matched against the external EMBL and NCBI databases. Our standard parameters for searching and scoring were set as follows: One missed cleavage site allowed. Fixed modifications: carbamidomethyl Cys. Variable modification: N-term. pyro-Glu, N-term. Gln, Met-oxidation. Peptide charge: 2+, 3+, 4+. Peptide tolerance: 1.0 Da. MS/MS tolerance: 0.8 Da. If not stated otherwise (see Results), a minimal score of 100 and/or minimal sequence coverage of 20% was set as cut-off for low-scoring hits.

### Sequence analysis

Basic sequence analysis was done using the LASERGENE software package from DNAstar (Madison, Wisconsin, USA). The IMG Data Management and Analysis platform (http://img.jgi.doe.gov) was used to inspect the *S. wolfei* genome and the identified genes, gene information and evidence of function prediction, as well as the positional cluster genes (gene clusters) and ortholog neighbourhood regions. Database searches were done using BLAST at the NCBI website, and the general domains and motifs in protein sequences were scanned in the NCBI Conserved Domain Database (CDD) search [50] and their domain architectures compared using the NCBI Conserved Domain Architecture Retrieval Tool (CDART) [51]. Transmembrane helices were scanned in the program TMHMM 2.0 and signal sequences for protein export in the programs SignalP 4.0 [52] and TatP 1.0 [53] at the Center for Biological Sequence Analysis of Technical University of Denmark (http://www.cbs.dtu.dk); PRED-TAT was also used for signal-peptide prediction [54].

## Supporting Information

Figure S1 **Two-dimensional Blue-Native/SDS-PAGE gel of solubilized membrane proteins of** ***S. wolfei*** **cells grown with butyrate and crotonate.**
(PDF)

Figure S2 **Membrane protein bands on SDS-PAGE gels that appeared only if the membranes had been solubilised with SDS, but not when dodecylmaltoside was used.**
(PDF)

Figure S3 **Time course of formate dehydrogenase-activity staining of membrane proteins separated by anoxic Blue-Native PAGE.**
(PDF)

Figure S4 **Time course of formate dehydrogenase-activity staining of soluble proteins separated by anoxic Blue-Native PAGE.**
(PDF)

Figure S5 **Time course of hydrogenase-activity staining of membrane proteins separated by anoxic Blue-Native PAGE.**
(PDF)

Figure S6 **Time course of hydrogenase-activity staining of soluble proteins separated by anoxic Blue-Native PAGE.**
(PDF)

Figure S7 **Formate dehydrogenase-activity staining of dodecylmaltoside-solubilised crude extract from butyrate-grown** ***S. wolfei*** **cells separated by anoxic Blue-Native PAGE.**
(PDF)

Figure S8 **Phylogenetic tree of the amino-acid sequence alignment of the EtfA and EtfB expressed in** ***S. wolfei*** **.**
(PDF)

Figure S9 **Amino-acid sequence alignment in an attempt to predict the ion specificity of the ATP synthase of** ***S. wolfei*** **.**
(PDF)

Table S1 **Identifications obtained by peptide fingerprinting-mass spectrometry for the protein bands excised from activity-stained gel strips.**
(PDF)

Table S2 **Identifications obtained by peptide fingerprinting-mass spectrometry for protein bands excised from an activity-stained gel strip after separation of dodecylmaltoside-solubilised crude extract.**
(PDF)
