# Supplementary material for: A Proteomic View at the Biochemistry of Syntrophic Butyrate Oxidation in Syntrophomonas wolfei
Source: PLoS One. 2013 Feb 26;8(2):e56905. doi: 10.1371/journal.pone.0056905 (PMC3582634; doi:10.1371/journal.pone.0056905)
Supplement: Figure S1 — Two-dimensional Blue-Native/SDS-PAGE gel of solubilized membrane proteins of S. wolfei cells grown with butyrate and crotonate. (PDF) [file pone.0056905.s001.pdf]

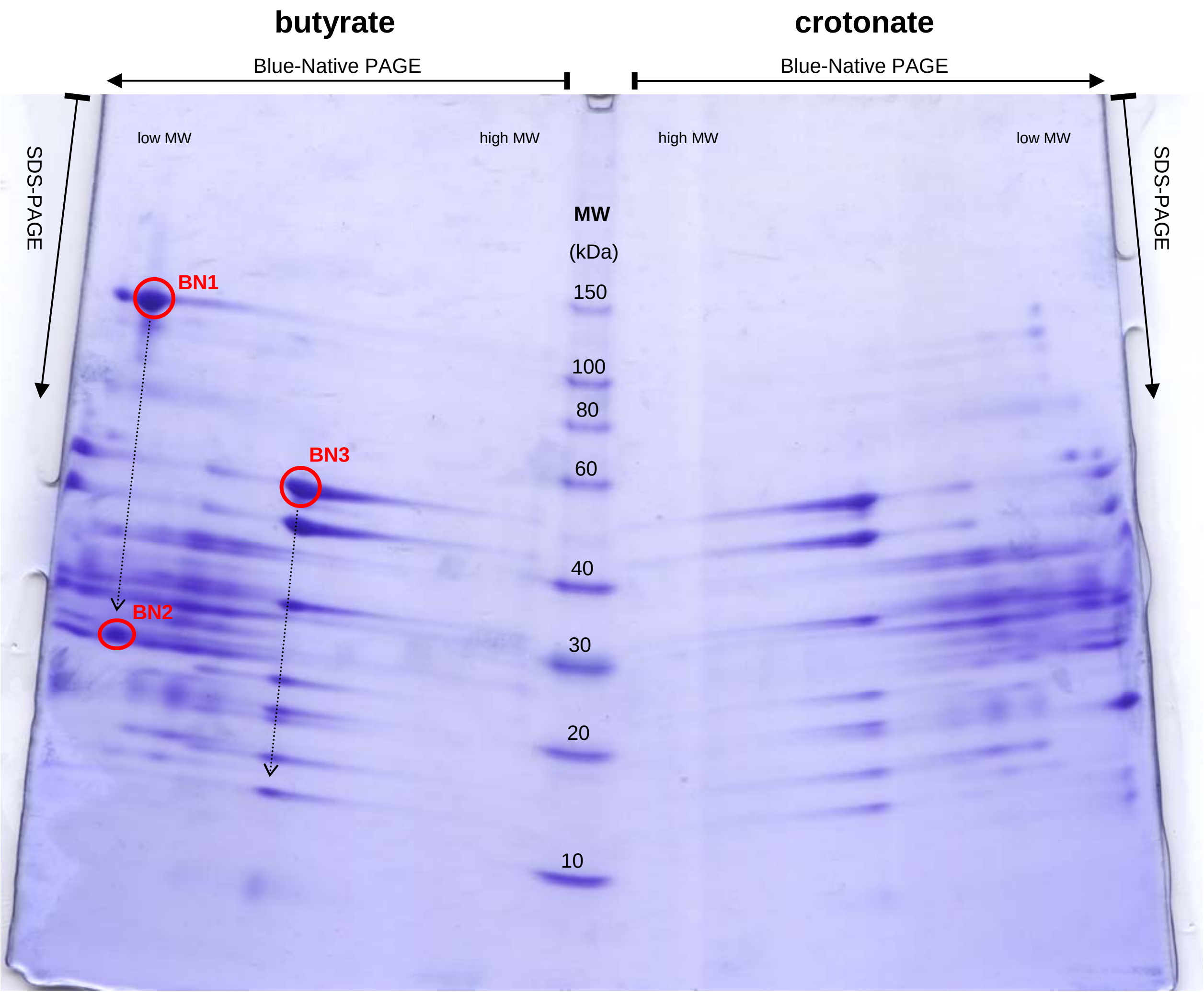

Fig. S1. Two-dimensional Blue-Native/SDS-PAGE gel of solubilized membrane proteins of *S. wolfei* cells grown with butyrate (left) and crotonate (right). Solubilised protein complexes were separated by non-denaturing Blue-Native PAGE in the first dimension (not shown). The gel strips from BN-PAGE were inserted (mirror-inverted) into a denaturating SDS-PAGE gel (gradient gel, 5 - 18% polyacrylamide) for separation of the subunits of the protein complexes in the second dimension. Protein spots excised from the SDS gel for identification by peptide fingerprinting-mass spectrometry are indicated (BN1 – BN3) and were attributed to the following genes in *S. wolfei* (see also main text): BN1, Swol_0800 and Swol_0799 annotated to conjointly encode a selenocysteine-linked formate dehydrogenase catalytic subunit (of the FDH-2 complex); BN2, Swol_0798 annotated as corresponding iron-sulfur subunit of the formate dehydrogenase FDH-2 complex; BN4, Swol_2384, large subunit of the proton/sodium-translocation ATP-synthase complex. MW, marker proteins with molecular masses indicated (in kDa).
