# Supplementary material for: A Proteomic View at the Biochemistry of Syntrophic Butyrate Oxidation in Syntrophomonas wolfei
Source: PLoS One. 2013 Feb 26;8(2):e56905. doi: 10.1371/journal.pone.0056905 (PMC3582634; doi:10.1371/journal.pone.0056905)
Supplement: Figure S2 — Membrane protein bands on SDS-PAGE gels that appeared only if the membranes had been solubilised with SDS, but not when dodecylmaltoside was used. (PDF) [file pone.0056905.s002.pdf]

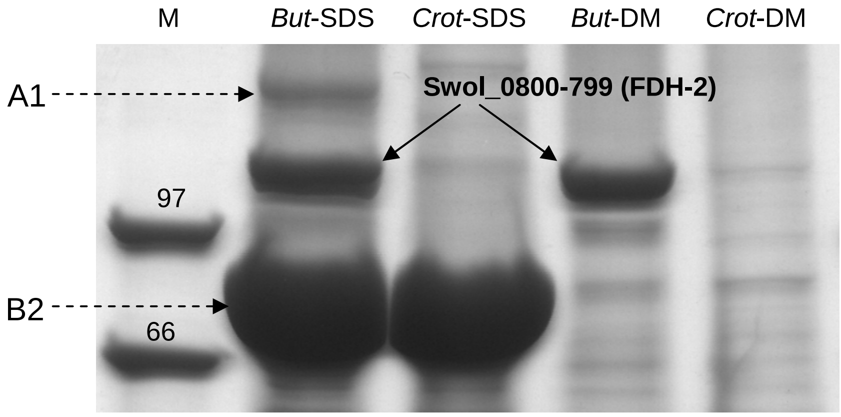

Fig. S2. Two membrane protein bands appeared on SDS-PAGE gels only if the membranes had been solubilised with SDS, but not when dodecylmaltoside was used. Legend: M, marker proteins with molecular mass indicated (in kDa); *But*, membrane proteins derived of butyrate-grown *S. wolfei*; *Crot*, membrane proteins derived of crotonate-grown *S. wolfei*; SDS, membrane proteins solubilised with SDS; DM, membrane proteins solubilised with dodecylmaltoside. Identifications for bands A1 and B2: see Table 1 in main text. The strong band of the FDH-2 subunit (Swol_0800-799) observed exclusively for butyrate-grown cells is also indicated (see text).
