# Supplementary material for: A Proteomic View at the Biochemistry of Syntrophic Butyrate Oxidation in Syntrophomonas wolfei
Source: PLoS One. 2013 Feb 26;8(2):e56905. doi: 10.1371/journal.pone.0056905 (PMC3582634; doi:10.1371/journal.pone.0056905)
Supplement: Figure S6 — Time course of hydrogenase-activity staining of soluble proteins separated by anoxic Blue-Native PAGE. (PDF) [file pone.0056905.s006.pdf]

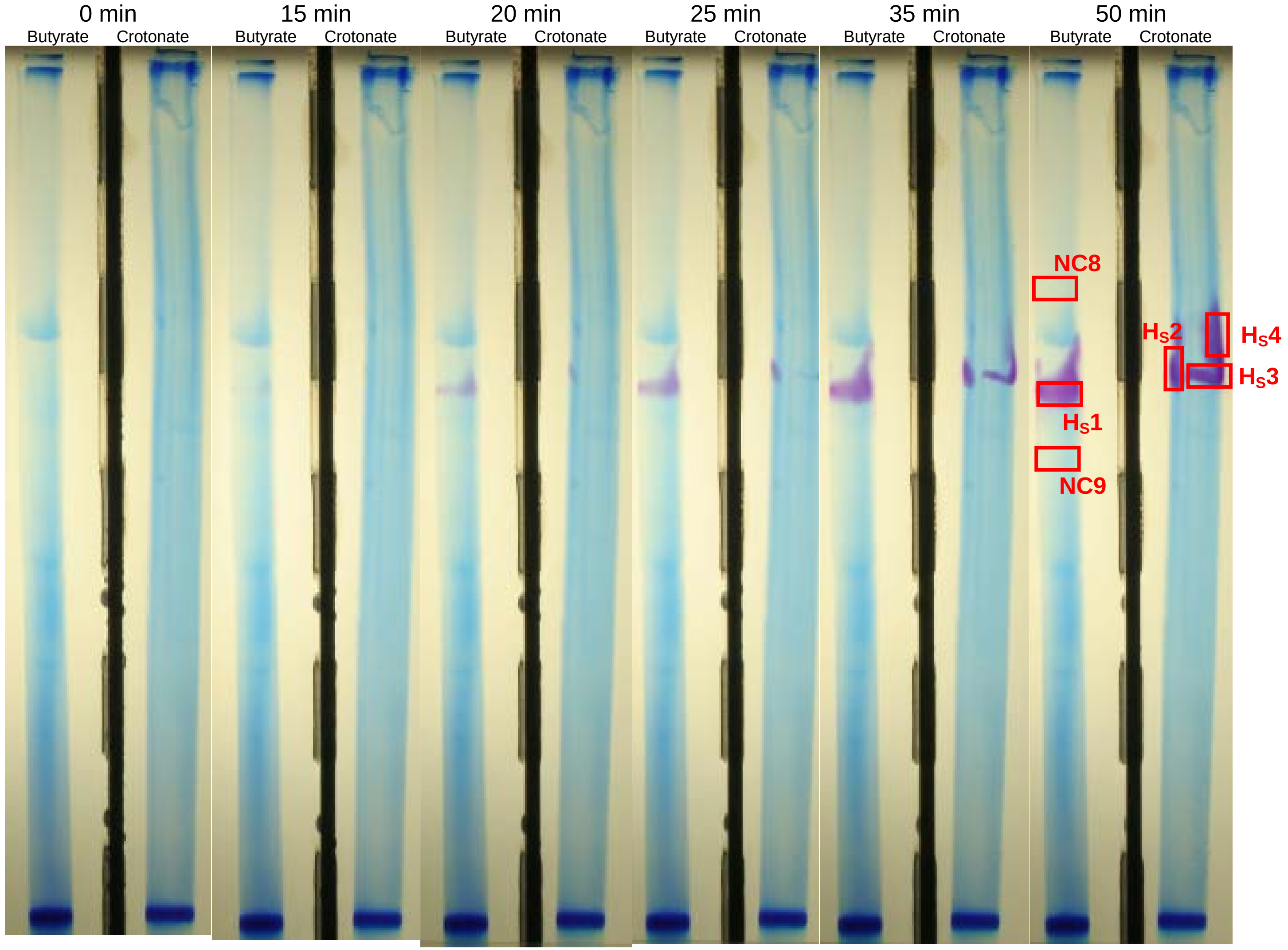

Fig. S6. Time course of hydrogenase-activity staining of soluble proteins separated by anoxic Blue-Native PAGE. Staining was perfomed with 1 mM benzyl viologen in 50 mM potassium phosphate buffer, pH 7.5, and started with 1 atm H_2.
