# Supplementary material for: A Proteomic View at the Biochemistry of Syntrophic Butyrate Oxidation in Syntrophomonas wolfei
Source: PLoS One. 2013 Feb 26;8(2):e56905. doi: 10.1371/journal.pone.0056905 (PMC3582634; doi:10.1371/journal.pone.0056905)
Supplement: Figure S7 — Formate dehydrogenase-activity staining of dodecylmaltoside-solubilised crude extract from butyrate-grown S. wolfei cells separated by anoxic Blue-Native PAGE. (PDF) [file pone.0056905.s007.pdf]

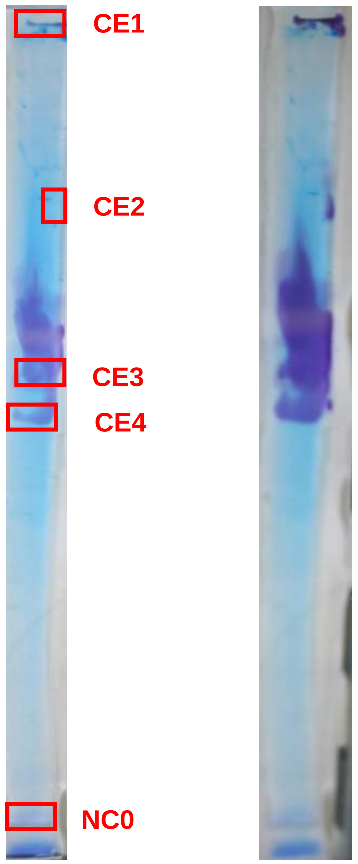

Fig. S7. Formate dehydrogenase-activity staining of dodecylmaltoside-solubilised crude extract from butyrate-grown *S. wolfei* cells separated by anoxic Blue-Native PAGE after 6 min (left) and 12 min (right). Staining was performed with 1 mM benzyl viologen in 50 mM potassium phosphate buffer, pH 7.5, and started by addition of 5 mM formate.
