# Supplementary material for: A Proteomic View at the Biochemistry of Syntrophic Butyrate Oxidation in Syntrophomonas wolfei
Source: PLoS One. 2013 Feb 26;8(2):e56905. doi: 10.1371/journal.pone.0056905 (PMC3582634; doi:10.1371/journal.pone.0056905)
Supplement: Figure S8 — Phylogenetic tree of the amino-acid sequence alignment of the EtfA and EtfB expressed in S. wolfei . (PDF) [file pone.0056905.s008.pdf]

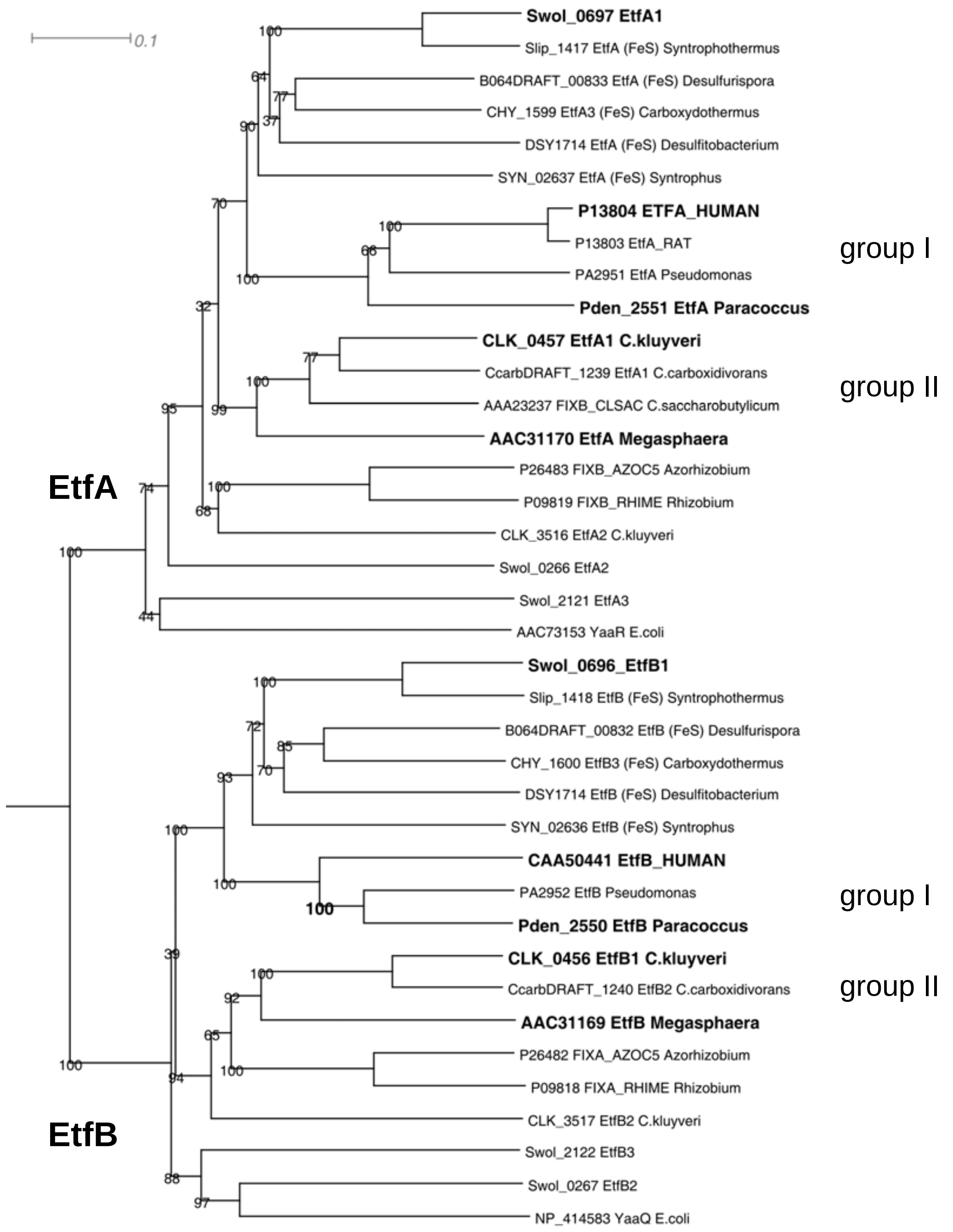

Fig. S8. Phylogenetic tree of the amino-acid sequence alignment of the EtfA (top) and EtfB (bottom) expressed in *S. wolfei* (Swol_0697-96, see main text) with representative sequences of EtfABs that coordinate either one FAD and one AMP (group I), or two FAD but no AMP (group II) [ref 1**].** Sequences of typical group-I EtfABs are from *Paracoccus denitrificans* and human mitochondria, and for group II from *Megasphaera elsdenii* and *Clostridium kluyveri* (indicated by bold letters). Note that Swol_0697-96 and the other EtfABs that are also encoded in a gene cluster with FeS-oxidoreductase (DUF224) in other organisms (all sequences labelled with 'FeS'), seem to be more closely related to the group-I EtfABs. Accession numbers or locus tags are indicated. The sequence alignment and the neighbor-joining tree building involved the CLUSTALX2 software and visualization of the tree the DENDROSCOPE software. The Rossmann-like *alpha*/*beta*/*alpha*-sandwich fold (IPR014729) containing protein P31663 of *E. coli* served as outgroup/root (not shown).

Ref. 1: Sato K, Nishina Y, Shiga K (2003) Purification of electron-transferring flavoprotein from *Megasphaera elsdenii* and binding of additional FAD with an unusual absorption spectrum. J Biochem 134: 719-729.
