# Supplementary material for: A Proteomic View at the Biochemistry of Syntrophic Butyrate Oxidation in Syntrophomonas wolfei
Source: PLoS One. 2013 Feb 26;8(2):e56905. doi: 10.1371/journal.pone.0056905 (PMC3582634; doi:10.1371/journal.pone.0056905)
Supplement: Figure S9 — Amino-acid sequence alignment in an attempt to predict the ion specificity of the ATP synthase of S. wolfei . (PDF) [file pone.0056905.s009.pdf]

	Organism	Alignment
H^+	*Escherichia coli*	-LAAIGAAIGIGILGGKFLEGAARQPDLIPLLRTQFFIVMGLV**D**AIPMI-
H^+	*Vibrio cholerae*	-LCAVGTAIGFAVLGGKFLEGAARQPEMAPMLQVKMFIIAGLL**D**AVPMI-
H^+	*Vibrio alginolyticus*	-LASLGTAIGFALLGGKFLEGAARQPEMAPMLQVKMFIIAGLL**D**AVPMI-
H^+	*Bacillus subtilis*	-LGALGAGIGNGLIVSRTVEGIARQPEAGKELRTLMFMGIALV**E**ALPII-
H^+	*Entherococcus hirae*	-GAAIGAGYGNGQVISKTIESMARQPEMSGQLRTTMFIGVALV**E**AVPIL-
Na^+	*Acetobacterium woodii c3*	-IAGVG**P**GIG**Q**GFAAGKGAEAVGRQPEAQSDIIRTMLLGAAVA**ETT**GIY-
Na^+	*Acetobacterium woodii c1*	-VAGVG**P**GIG**Q**GFAAGKGAEAVGKNPTKSNDIVMIMLLGAAVA**ETS**GIF-
Na^+	*Propionigenium modestum*	-IAGIG**P**GVG**Q**GYAAGKAVESVARQPEAKGDIISTMVLGQAIA**EST**GIY-
Na^+	*Thermotoga maritima*	-IGAIG**P**GIG**E**GNIGAHAMDAMARQPEMVGTITTRMLLADAVA**ETT**GIY-
	Syntrophomonas wolfei	-IAGIG**G**GIG**M**GIAGGKAFEAIARQPEVGGDVRTLLFITLAFI**ETL**TIY-

Fig. S9. Amino-acid sequence alignment according to Dzioba *et al.* [1] in an attempt to predict the ion specificity of the ATP synthase of *S. wolfei* (gene cluster Swol_2381-Swol_2388). The amino-acid sequence motive of ATP synthase c-subunits that likely predicts Na^+-binding, **P**-x_3-**Q**-x_{32}-**ET** [see refs. 1 and 2] is not conserved in the *S. wolfei* sequence of Swol_2387.

References for Supplemental information Fig. S9:

1. Dzioba J, Häse CC, Gosink K, Galperin MY, Dibrov P (2003) Experimental verification of a sequence-based prediction: F_1F_0-type ATPase of *Vibrio cholerae* transports protons, not Na^+ ions. J Bacteriol 185: 674-678.
2. Rahlfs S, Müller V (1997) Sequence of subunit c of the Na^+-translocating F_1F_0 ATPase of *Acetobacterium woodii*: proposal for determinants of Na^+ specificity as revealed by sequence comparisons. FEBS Letters 404: 269-271.
