# Supplementary material for: A Proteomic View at the Biochemistry of Syntrophic Butyrate Oxidation in Syntrophomonas wolfei
Source: PLoS One. 2013 Feb 26;8(2):e56905. doi: 10.1371/journal.pone.0056905 (PMC3582634; doi:10.1371/journal.pone.0056905)
Supplement: Table S1 — Identifications obtained by peptide fingerprinting-mass spectrometry for the protein bands excised from activity-stained gel strips. (PDF) [file pone.0056905.s010.pdf]

Tab. S1. Identifications obtained by peptide fingerprinting-mass spectrometry for the protein bands excised from activity-stained gel strips (see Figs. S3 – S6). The formate dehydrogenase catalytic subunit FDH-2 was evaluated as fusion protein (Swol_0800 sequence followed by selenocysteine-linked Swol_0799 sequence). Numbering of FDH-1 – FDH-5 and HYD-1 – HYD-3 catalytic subunits in the genome sequence of *S. wolfei* according to ref. 1.

Spot No.	Locus Tag (Swol_)	Annotation	Predicted mass (Da)	Score	Seq. coverage (%)
FDH_M1	0133	hypothetical outer-membrane protein (N-terminal copper-amine oxidase domain)	78,098	325	39
	2382	ATP synthase, proton/sodium translocating, F1 *beta*-subunit (COG0055)	51,311	110	13
	1934	acetyl-CoA acetyltransferase (COG0183)	41,282	109	29
	1630	ABC-type transport permease protein (tungstate uptake)	38,493	61	13
FDH_M1	1934	acetyl-CoA acetyltransferase (COG0183)	41,282	89	14
	**0800-799**	**formate dehydrogenase catalytic subunit (FDH-2)**	122,381	87	31
	1630	ABC-type transport permease protein (tungstate uptake)	38,493	79	9
	0133	hypothetical outer-membrane protein (N-terminal copper-amine oxidase domain)	78,098	69	17
	0331	TRAP-type transport substrate-binding protein (COG1638)	41,235	63	4
	0413	conserved hypothetical lipoprotein	42,747	59	9
FDH_S	1029	NADH dehydrogenase I chain G	39,333	63	16
	**1028**	**formate dehydrogenase catalytic subunit (FDH-3)**	59,637	35	11
H_M1	**0800-799**	**formate dehydrogenase catalytic subunit (FDH-2)**	122,381	135	14
	1630	ABC-type transport permease protein (tungstate uptake)	38,493	91	13
	0331	TRAP-type transport substrate-binding protein (COG1638)	41,235	53	9
	0413	conserved hypothetical lipoprotein	42,747	45	11
H_M2	1630	ABC-type transport permease protein (tungstate uptake)	38,493	288	39
	2556	ABC-type transport substrate-binding protein (amino acid uptake)	41,858	83	16
	1934	acetyl-CoA acetyltransferase (COG0183)	41,282	80	23
	1924	hypothetical protein	74,129	28	28
H_M3	2383	ATP synthase, proton/sodium translocating, F1 *gamma*-subunit	33,080	99	28
	**1925**	**hydrogenase catalytic subunit (HYD-2)**	41,807	82	14
	0413	conserved hypothetical lipoprotein	42,747	74	16
	2384	ATP synthase, proton/sodium translocating, F1 *alpha*-subunit	54,508	71	12

H_M4	2382	ATP synthase, proton/sodium translocating, F1 *beta*-subunit	51,311	106	18
	2384	ATP synthase, proton/sodium translocating, F1 *alpha*-subunit	54,508	104	17
	**1925**	**hydrogenase catalytic subunit (HYD-2)**	41,807	97	15
	0698	iron-sulfur membrane protein (cysteine-rich DUF224 protein)	81,417[e]	50	12
H_S1	1934	acetyl-CoA acetyltransferase (COG0183)	41,282	438	32
	2054	putative flavoprotein	98,399	75	17
H_S2	1934	acetyl-CoA acetyltransferase (COG0183)	41,282	67	17
	1933	butyryl-CoA dehydrogenase (COG1960)	67,829	63	20
	1850	3-oxoacyl-(acyl-carrier protein) reductase	27,226	46	18
	0768	acetate kinase (COG0282)	43,419	32	19
	**1017**	**hydrogenase catalytic subunit (HYD-1)**	62,988	29	8
H_S3	1934	acetyl-CoA acetyltransferase (COG0183)	41,282	117	27
	1933	butyryl-CoA dehydrogenase (COG1960)	67,829	40	30
	0768	acetate kinase (COG0282)	43,419	25	25
	0697	electron transfer flavoprotein, *alpha*-subunit (EtfA)	33,042	24	13
	**1017**	**hydrogenase catalytic subunit (HYD-1)**	62,988	22	10
H_S4	1934	acetyl-CoA acetyltransferase (COG0183)	41,282	72	18
	**1017**	**hydrogenase catalytic subunit (HYD-1)**	62,988	26	9
NC1	0331	TRAP-type transport substrate-binding protein (COG1638)	41,235	64	10
	0413	conserved hypothetical lipoprotein	42,747	60	15
	1934	acetyl-CoA acetyltransferase (COG0183)	41,282	56	30
NC2	1934	acetyl-CoA acetyltransferase (COG0183)	41,282	54	21
	2384	ATP synthase, proton/sodium translocating, F1 *alpha*-subunit	54,508	46	11
NC3	1935	3-hydroxybutyryl-CoA dehydrogenase (COG1250)	29,761	140	28
	1934	acetyl-CoA acetyltransferase (COG0183)	41,282	70	33
NC4	0468	alanine-tRNA ligase	98,157	97	10
	1017	hydrogenase catalytic subunit (HYD-1)	62,988	85	19
	0768	acetate kinase (COG0282)	43,419	56	33

NC5	1934	acetyl-CoA acetyltransferase (COG0183)	41,282	80	31
	0768	acetate kinase (COG0282)	43,419	65	16
	0468	Alanine-tRNA ligase	98,157	53	14
NC6	1630	ABC-type transport permease protein (tungstate uptake)	38,493	104	26
	2384	ATP synthase, proton/sodium translocating, F1 *alpha*-subunit	54,508	77	12
	2382	ATP synthase, proton/sodium translocating, F1 *beta*-subunit	51,311	45	20
NC7	1934	acetyl-CoA acetyltransferase (COG0183)	41,282	156	41
	1630	ABC-type transport permease protein (tungstate uptake)	38,493	95	31
	2382	ATP synthase, proton/sodium translocating, F1 *beta*-subunit	51,311	89	26
NC8	1935	3-hydroxybutyryl-CoA dehydrogenase (COG1250)	29,761	132	11
	2382	ATP synthase, proton/sodium translocating, F1 *beta*-subunit	51,311	66	18
	1934	acetyl-CoA acetyltransferase (COG0183)	41,282	52	17
NC9	1933	butyryl-CoA dehydrogenase (COG1960)	67,829	333	32
	0768	acetate kinase (COG0282)	43,419	68	17

Reference for Supplemental information Table S1 :

1. Sieber JR, Sims DR, Han C, Kim E, Lykidis A, et al. (2010) The genome of *Syntrophomonas wolfei*: new insights into syntrophic metabolism and biohydrogen production. Environ Microbiol 12: 2289-2301.
