# Supplementary material for: A Proteomic View at the Biochemistry of Syntrophic Butyrate Oxidation in Syntrophomonas wolfei
Source: PLoS One. 2013 Feb 26;8(2):e56905. doi: 10.1371/journal.pone.0056905 (PMC3582634; doi:10.1371/journal.pone.0056905)
Supplement: Table S2 — Identifications obtained by peptide fingerprinting-mass spectrometry for protein bands excised from an activity-stained gel strip after separation of dodecylmaltoside-solubilised crude extract. (PDF) [file pone.0056905.s011.pdf]

Table S2. Identifications obtained by peptide fingerprinting-mass spectrometry for protein bands excised from an activity-stained gel strip after separation of dodecylmaltoside-solubilised crude extract (see Fig. S7). The formate dehydrogenase catalytic subunits FDH-2 and FDH-1 were evaluated as fusion proteins (selenocysteine-linked). Numbering of FDH-1 and FDH-2 catalytic subunits in the genome sequence of *S. wolfei* according to ref. 1.

Spot No.	LocTag (Swol_)	Annotation	Predicted mass (Da)	Score	Seq. coverage (%)
CE1	0143	hypothetical outer membrane protein (invasin/intimin/lectin-like)	141,664	159	15
	1945	Putative activator of (R)-2-hydroxyglutaryl-coA dehydratase	159,565	111	40
	**0800-799**	**formate dehydrogenase catalytic subunit (FDH-2)**	95,579	100	10
CE2	**0800-799**	**formate dehydrogenase catalytic subunit (FDH-2)**	95,579	115	19
	2030	3-hydroxybutyryl-CoA dehydrogenase	29,998	100	55
CE3	1934	acetyl-CoA acetyltransferase	41,282	285	63
	**1017**	**hydrogenase catalytic subunit (HYD-1)**	62,988	154	23
	0384	uncharacterized protein	68,239	84	16
	**0785-86**	**formate dehydrogenase catalytic subunit (FDH-1)**	58,258	82	19
CE4	1934	acetyl-CoA acetyltransferase	41,282	309	54
	**0785-86**	**formate dehydrogenase catalytic subunit (FDH-1)**	58,258	90	13
NC0	1934	acetyl-CoA acetyltransferase	41,282	68	23

[ref. 1: Sieber JR, Sims DR, Han C, Kim E, Lykidis A, et al. (2010) The genome of *Syntrophomonas wolfei*: new insights into syntrophic metabolism and biohydrogen production. Environ Microbiol 12: 2289-2301]
